# Expression and Manipulation of the APC-β-Catenin Pathway During Peripheral Neuron Regeneration

**DOI:** 10.1038/s41598-018-31167-1

**Published:** 2018-09-04

**Authors:** Arul Duraikannu, Jose A. Martinez, Ambika Chandrasekhar, Douglas W. Zochodne

**Affiliations:** 1 Division of Neurology, Department of Medicine & Neuroscience and Mental Health Institute, University of Alberta, Edmonton, Alberta, Canada; 2 Formerly of Hotchkiss Brain Institute and Department of Clinical Neurosciences, University of Calgary, Calgary, Alberta Canada

## Abstract

Molecules and pathways that suppress growth are expressed in postmitotic neurons, a potential advantage in mature neural networks, but a liability during regeneration. In this work, we probed the APC (adenomatous polyposis coli)-β-catenin partner pathway in adult peripheral sensory neurons during regeneration. APC had robust expression in the cytoplasm and perinuclear region of adult DRG sensory neurons both before and after axotomy injury. β-catenin was expressed in neuronal nuclei, neuronal cytoplasm and also in perineuronal satellite cells. In injured dorsal root ganglia (DRG) sensory neurons and their axons, we observed paradoxical APC upregulation, despite its role as an inhibitor of growth whereas β-catenin was downregulated. Inhibition of APC in adult sensory neurons and activation of β-catenin, LEF/TCF transcriptional factors were associated with increased neuronal plasticity *in vitro*. Local knockdown of APC, at the site of sciatic nerve crush injury enhanced evidence for electrophysiological, behavioural and structural regeneration *in vivo*. This was accompanied by upregulation of β-catenin. Collectively, the APC-β-catenin-LEF/TCF transcriptional pathway impacts intrinsic mechanisms of axonal regeneration and neuronal plasticity after injury, offering new options for addressing axon regeneration.

## Introduction

Despite misconceptions that their regrowth is robust, the axons of peripheral neurons experience only slow and incomplete recovery following injury by trauma or disease. Hesitant early outgrowth, limited intrinsic growth properties, misdirection and a hostile microenvironment all conspire to render ‘permanent’ neurological deficits in patients. New approaches to engage a more robust growth potential in neurons are required.

Manipulating the intrinsic properties of neurons and providing them with an accelerated growth pattern at the outset of regeneration is a newer approach toward improving these outcomes. However, to identify candidate proteins and pathways with the potential to significantly alter growth in otherwise stable, connected adult neurons, lessons from tumour biology are of interest. Among the repertoire of oncology molecules, including those robustly expressed in adult neurons, some attenuate cellular growth as ‘tumour suppressor’ molecules. PTEN (phosphatase and tensin homolog deleted on chromosome ten) and Rb1 (retinoblastoma) are examples that act as ‘brakes’ to neuronal regenerative regrowth^1,2^. In turn, their knockdown has been associated with heightened growth in both naive and preconditioned adult neurons.

The Wnt signaling pathway, widely expressed, is of importance in peripheral and central neurons^3^. It has, for example, been linked to neurogenesis, neuron positioning, axon and dendrite development, synaptogenesis and myelin compaction^4,5^. Additional, roles of Wnt signaling have been identified in axon guidance of corticospinal tract (CST) axons^6^ and in neuroprotection and regeneration after optic nerve injury^7^.

Specific molecules within this pathway include the tumor suppressor gene adenomatous polyposis coli (APC), frequently mutated in colorectal cancer^8^. APC operates to impede the key transcriptional molecule of the Wnt pathway, namely β-catenin by participating in its destruction complex, forming a structural scaffold to phosphorylate β-catenin. Phosphorylated β-catenin is subsequently degraded by the proteasome^9^. Loss of wild type APC expression results in the nuclear accumulation of β-catenin, which interacts with TCF/LEF transcription factors to cause aberrant gene transcription and cell proliferation. For example, over-expression of β-catenin results in enhanced tumour cell proliferation, increased cell motility and resistance to apoptosis^10,11^.

β-catenin is important for neurons during development influencing neural proliferation, neuronal differentiation, dendrite formation and participating in the initial formation of the neural plate and neural crest^6,12–14^. For example, conditional ablation of β-catenin in neural stem cells and their progeny impair dentate gyrus neurogenesis^15,16^. In later stages it has roles in the mature central nervous system, including regulation of the neuronal cytoskeleton and synaptic differentiation^13^, axon guidance and neuronal cell survival^17^. β-catenin phosphorylation at residue Y654 and Y142 increased axon growth and branching through TCF4/β-catenin-dependent transcription in hippocampal neurons *in vitro*^12^. Wnt/β-catenin is activated in the spinal cord in models of neuropathic pain^18,19^. Galectin-3 knockout mice expressed higher levels of β-catenin, associated with enhanced neuronal survival, axon regeneration and recovery of motor function in reinnervated hind limbs after traumatic nerve lesions^20^. Moreover, β-catenin is required for Schwann cell proliferation *in vitro*, a key partnering step in nerve regeneration^20^.

Given the critical roles of β-catenin in the developing and adult nervous system it is an expectation that APC might impact them^21^. As expected through its interactions, APC influences proliferation, apoptosis, cell adhesion, and migration^7^. APC is strongly expressed in both the developing and adult nervous system^22^. APC has been detected in neurites of neuroblastoma cells^23^, dorsal root ganglion neurons^24^and cortical neurons^23^. Roles identified to date include cell cycle control of neuronal PC12 (pheochromocytoma) cells^25^, apoptosis of neural crest cells^26^, neurite formation^25^, and receptor accumulation in synapses^27^.

Collectively these findings provide a rationale to evaluate whether the activation of the β-catenin signal transduction pathway through APC manipulation promotes axon regeneration in adult neurons. Some work, however, has also suggested otherwise, identifying no impact of pre-injury β-catenin DRG knockdown on regeneration after nerve crush^28^. In this work we examined this pathway from a differing perspective through knockdown of the APC inhibitory interaction with β-catenin. Not previously examined, we confirm expression of key elements of the pathway in adult peripheral neurons. Moreover, by manipulating this pathway we identify an impact of APC knockdown on neuronal regrowth both *in vitro* and during regrowth *in vivo*. Inappropriate activation of APC signalling in injured adult neurons appears to offer a new hitherto unrecognized regenerative ‘brake’.

## Results

### Upregulation of APC gene expression in adult DRG sensory neurons following sciatic nerve axotomy

**Figure 1:**
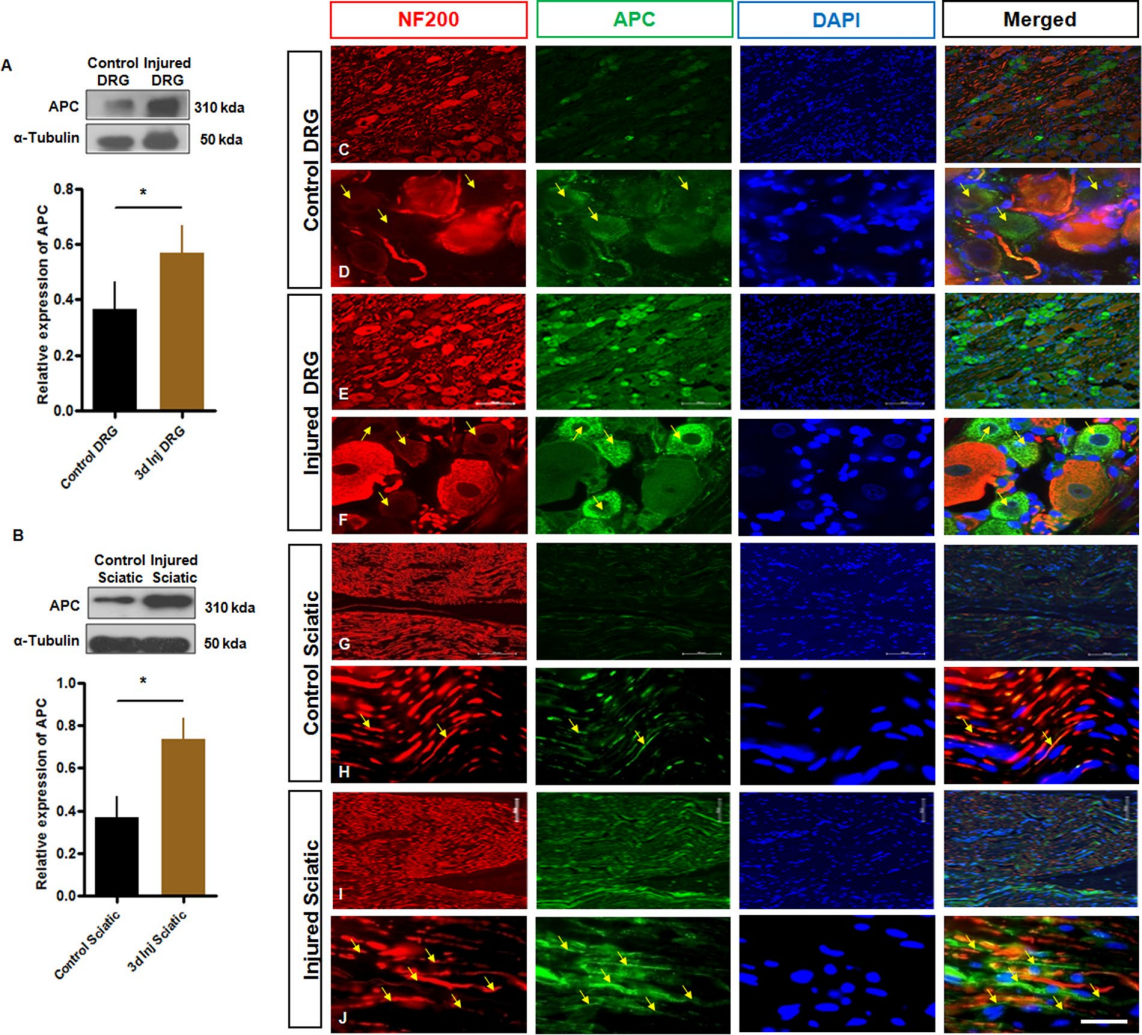
Expression and cellular distribution of APC protein in rat DRG and sciatic nerve after injury. (**A**,**B**) Western blot indicates the expression of APC (310 kDA) and α-tubulin (50 kDA) in control and 3 day injury DRG and sciatic nerve tissues. (**C**–**F**) Immunohistochemical analysis with anti-NF200 (red), APC (green), DAPI (blue) of the DRG, showing that APC is localized in sensory neurons with intense expression the small caliber neurons, respectively and that its apparent expression is increased at 3 days postaxotomy. (**D**,**F**) Higher magnification of DRG labeled with APC. Arrows show more intense expression of APC in smaller neurofilament poor DRG neurons. (**G**–**J)** Immunohistochemical analysis with anti-NF200 (red), APC (green), DAPI (blue) of the proximal sciatic nerve segments, showing that APC is expressed on regenerating axons and a limited number of the SCs at 3 days post axotomy. (**H**,**J**) Higher magnification of sciatic nerve labeled with APC. Arrows indicate the expression of APC in regenerating axons. Scale bars = 100 µm. (*p = Student’s t-test, p < 0.05, n = 3, mean ± SEM).

**Figure 2:**
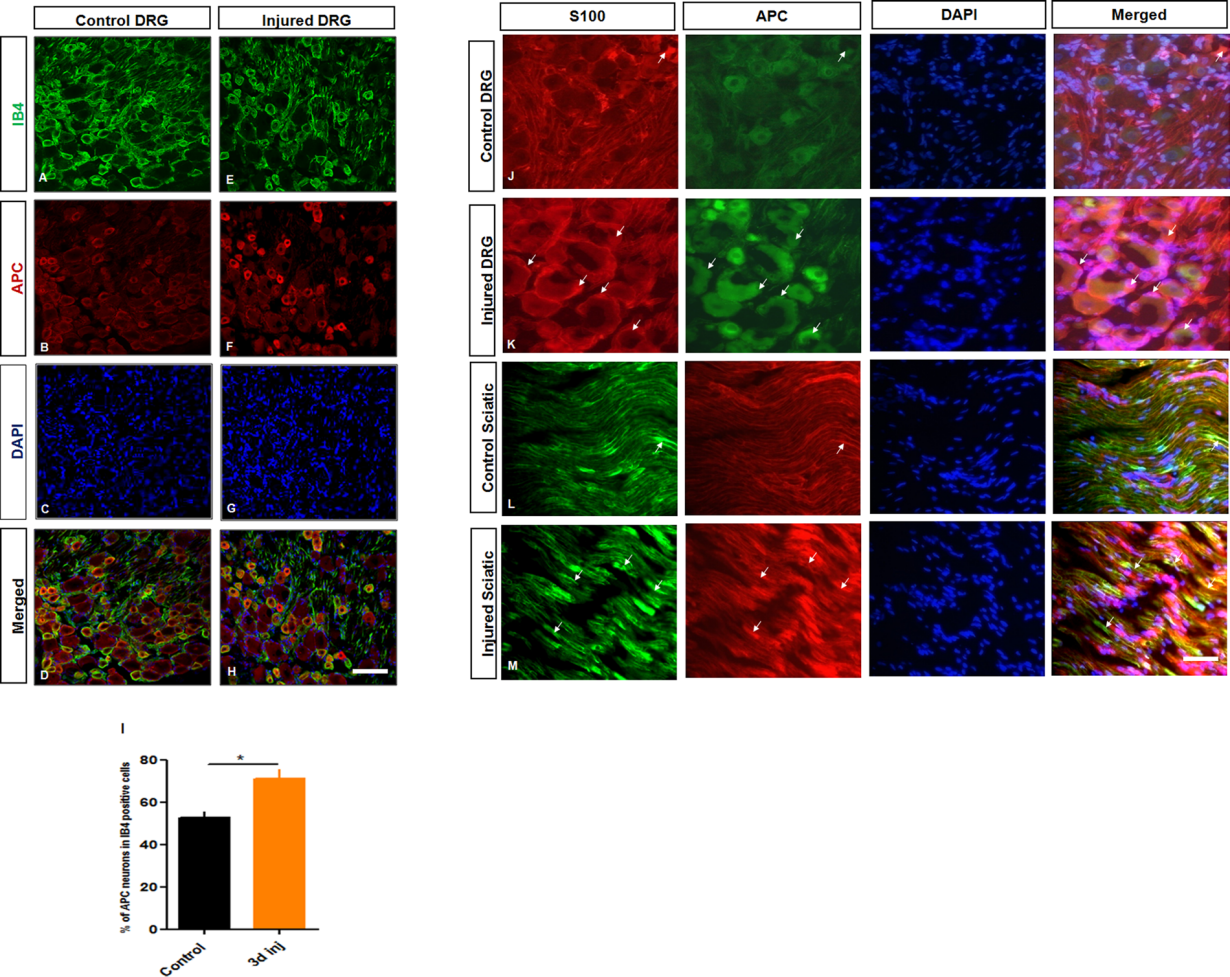
Immunohistochemical analysis of APC distribution and colocalization in the IB4 DRG neurons before and after rat sciatic nerve axotomy. (**A**–**H**) Representative images of the control and injured DRG, using the indicated antibodies; IB4 (Green), APC (red), DAPI (blue). Scale bar = 100 µm. APC was prominently, although not exclusively expressed on small nonpeptidergic neurons, especially after injury with robust expression in the cytoplasm and perinuclear region of DRG neurons *in vivo*. (**I**) Quantification of (**A**–**H**) showing APC expression in IB4 positive neurons in control and 3 d injured DRG. (**J**–**M**) Immunohistochemical analysis of APC expression and localization in DRG neurons, sciatic nerve and Schwann cells of control and injured rats. (**J**–**M**) Co-labeling of S100 (a marker for Schwann cells), APC and DAPI in both control and injured DRG and sciatic nerve indicates a degree of localization of APC in SCs. Scale bar = 100 µm. (*t*-test, *p < 0.05, n = 3, means ± SEM).

To examine the distribution of APC protein in normal or injured nerve tissue, we examined transverse cryosections of immunostained DRGs and sciatic nerves coupled with Western immunoblotting. Previous data have suggested that sensory neuron growth cones express APC^29^. There were increases in total APC protein expression in both DRG and sciatic nerve 3 d following injury (Fig. 1A,B). APC immunostaining was present in normal DRG sensory neurons, colabelling with NF200 (a marker for neurofilament) (Fig. 1C,D) and spinal motor neurons (Supplemental Fig. 1). Interestingly, APC presence was more prominently expressed in smaller caliber neurofilament poor DRG neurons (Fig. 1C,D) and there was lesser expression in Schwann cells (SCs) colabelled with S100 (a marker for Schwann cells) (Fig. 2J,L). In normal sciatic nerve, APC was expressed largely in axons(Fig. 1G,H) but with modest colabeling of SCs (Fig. 2L,M). Three days after transection, the intensity of APC expression increased in DRG neurons (Fig. 1E,F), motor neurons (Supplemental Fig. 1) and sciatic nerve (Fig. 1I,J). At the same time, expression of APC was maintained in glial cells after injury (Fig. 2K,M). Within the DRG after injury APC was widely expressed in the cytoplasm and perinuclear region, with ongoing prominent labeling of smaller caliber DRG sensory neurons (Fig. 1E,F), a pattern resembling that of PTEN^1^. The subclass of small caliber neurons with intense APC expression had marked colabeling with IB4 (binding the lectin Griffonia simplicifolia IB4; a marker of small, unmyelinated neurons) (Fig. 2A–H). After sciatic injury, the percentage of neurons expressing APC in IB4 labeled DRG neurons increased (Fig. 2I). APC was robustly expressed in regenerating axons following injury (Fig. 1I,J) in the proximal nerve stump. There was moderate expression of SC specific localization of APC at the regenerative nerve stump (Fig. 2M).

APC immunocytochemistry reactivity and protein expression by immunoblotting were also identified in dissociated and cultured DRG neurons after axotomy at 3d (Supplemental Fig. 2A–I). As above, its expression was more intense after injury in small DRG neurons (Supplemental Fig. 2E–H). Immunoblotting experiments further confirmed the upregulation of APC expression in cultured DRG neurons (Supplemental Fig. 2I). In the normal dissociated DRG cultures,IB4-positive small neurons were estimated to comprise approximately 40% of total neurons (Supplemental Fig. 3). Taken together these results indicate that APC, a potential growth barrier, paradoxically rises following injury and in a remarkable parallel to PTEN expression^1^, is very prominently expressed in slower growing nonpeptidergic IB4 binding neurons^30^.

### Nuclear β-catenin expression declines in sensory neurons following axotomy

**Figure 3:**
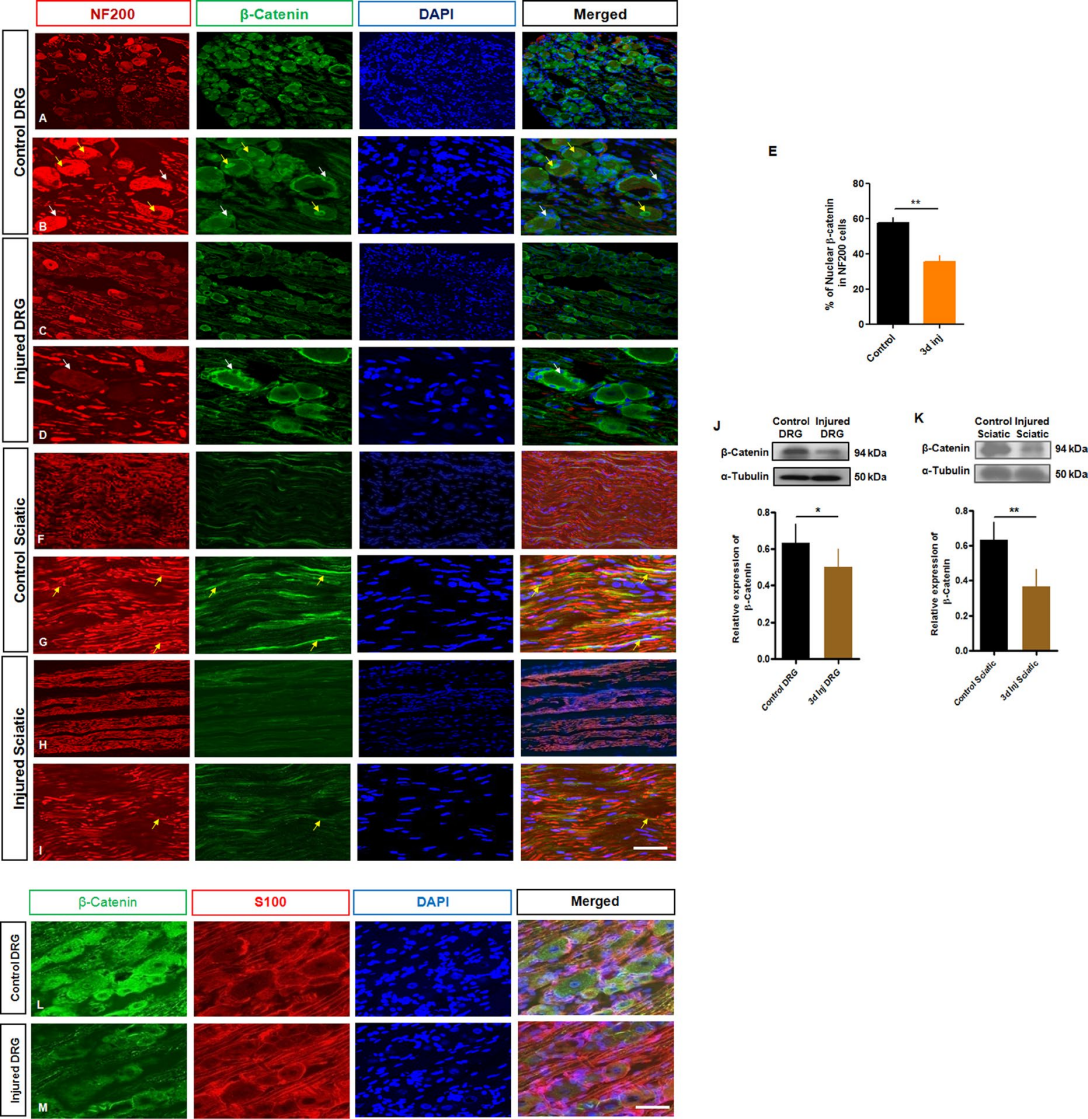
Axotomy injury reduces β-catenin protein expression in rat DRG and sciatic nerve. (**A**–**D**) Immunohistochemical analysis with anti-NF200 (red), APC (green), DAPI (blue) of the DRG, showing that β-catenin expression decreased in neuronal nuclei, neuronal cytoplasm and perineurial satellite cells, demonstrating reciprocal changes in comparison to those of APC following injury and at 3 days post axotomy. (**B**,**D**) Higher magnification of DRG labeled with β-catenin. Yellow arrows show the nuclear expression of β-catenin. White arrows indicate the expression of β-catenin in perineurial satellite cells (**E**). Quantification of nuclear APC expression in IB4 positive neurons in control and 3 d injured DRG. (**F**–**I**) Immunohistochemical analysis with anti-NF200 (red), β-catenin (green), DAPI (blue) of the proximal sciatic nerve segments, showing that β-catenin is expressed on some of the regenerating axons and SCs (Fig. 3G, arrow). (**G**,**I**) Higher magnification of sciatic nerve labeled with β-catenin. Arrows show the expression of β-catenin in regenerating axons. (**J**,**K**) Western blot analysis of β-catenin (310 kDA) and α-tubulin (50 kDA) in control and 3 day injured DRG and sciatic nerve. (**L**,**M**) Immunohistochemical analysis of β-catenin distribution in rat DRG neurons and satellite cells of intact and injured DRG *in vivo*. Representative images of β-catenin expression using the indicated antibodies; S100 (red), β-catenin (Green), with counterstaining of nuclei with DAPI (blue) shows an apparent reduction in the expression of β-catenin in satellite cells after injury. Scale bars = 100 µm. (*t*-test, *p < 0.05,**p < 0.01, n = 3, mean ± SEM).

**Figure 4:**
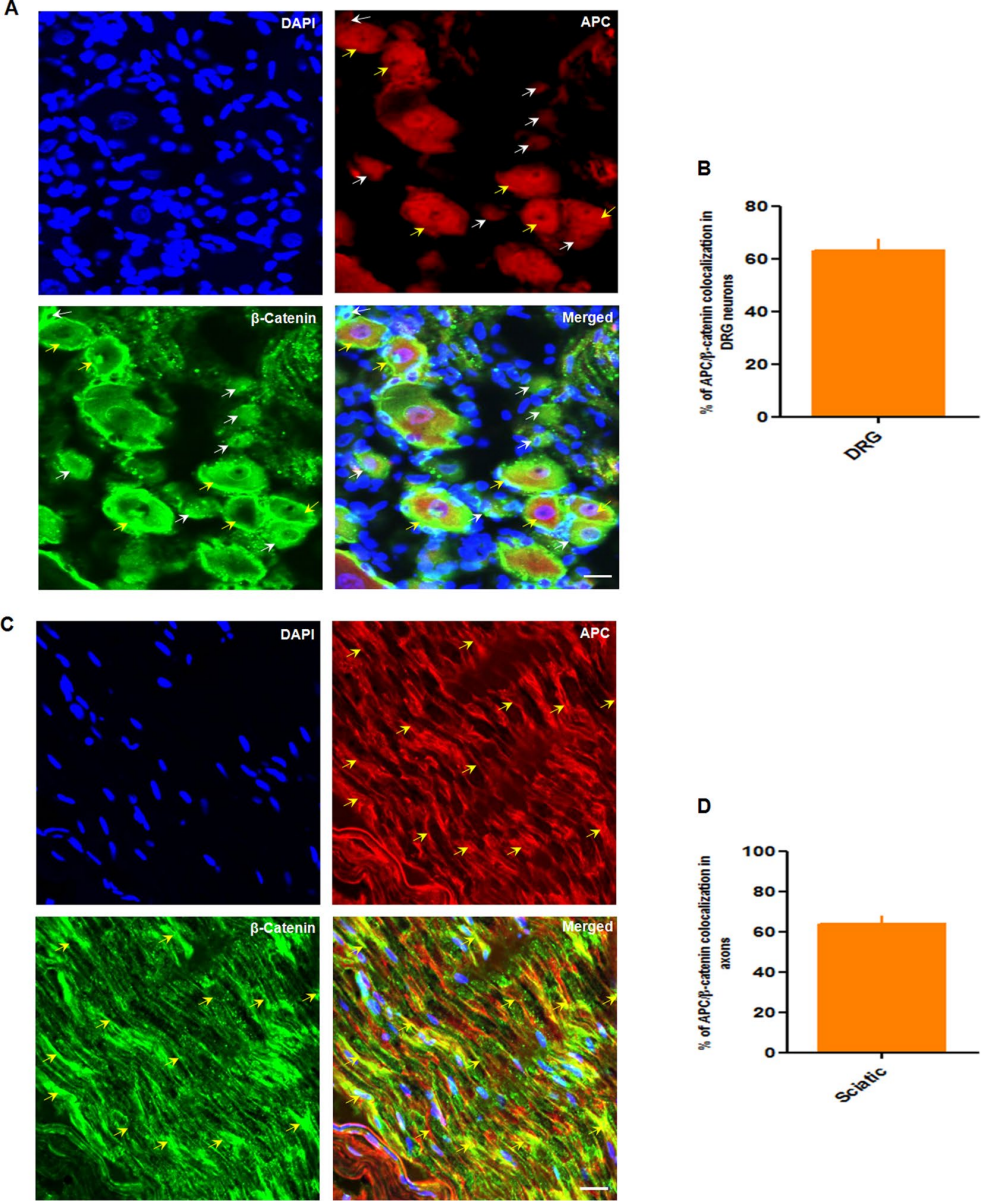
Transverse section of intact uninjured DRG and sciatic nerve with co-labeling of APC and β-catenin. (**A**–**D**) Immunohistochemistry of APC (red) and β-catenin (green). (**A**) APC is expressed in β-catenin positive DRG neurons however it expressed within most of the small (white arrow) and medium size (yellow arrow) neurons. (**B**) Quantification of A showing APC expression in β-catenin neurons in DRG (n = 4). (**C**) Immunohistochemistry of a longitudinal nerve section from an uninjured sciatic nerve. APC (red, yellow arrow) and β-catenin (green, yellow arrow) colocalize. (**D**) Quantification of C showing APC expression in β-catenin positive axons in the sciatic nerve (n = 3), indicating APC and β-catenin proteins are binding partners in DRG and sciatic nerve. Scale bar = 100 µm.

Given the close relationship between APC and its canonical downstream partner β-catenin, we examined the latter’s expression and localization in relation to injury. Immunohistochemistry of uninjured L4 and L5 DRGs showed that β-catenin was expressed in almost all sensory neuronal nuclei, neuronal cytoplasm and perineurial satellite cells (Fig. 3A–D), Supplemental Fig. 4. Interestingly, neurofilament labelling, which especially recognizes the medium and large category of DRG neurons, colabelled with β-catenin. Small IB4-positive neurons also prominently expressed β-catenin (Supplemental Fig. 4). β-catenin was co-expressed with the Schwann cell marker S100 in control and injured DRG (Fig. 3L,M). After injury however β-catenin expression in neuronal nuclei (Fig. 3C–E), satellite cells (Fig. 3M) and sciatic nerve appeared to decline (Fig. 3F–I). This was confirmed by western immunoblot from DRG and sciatic nerves (Fig. 3J,K). APC is known to interact with β-catenin and tag it for proteasomal degradation. To determine if there is a spatial relationship between these partners their colabelling was explored in DRG sensory neurons and sciatic nerve. APC was indeed co-expressed with β-catenin indicating their combined localization in both small and medium size DRG neurons and sciatic nerve (Fig. 4A–D). β-catenin immunohistochemical expression also appeared to decline in dissociated DRG neuronal cells and in their neurites following injury at day 3 *in vitro* (Supplemental Fig. 5A–H). Declines were confirmed by western immunoblot (Supplemental Fig. 5I). These findings identified a reciprocal relationship between the expression of APC and β-catenin, with declines in the latter after axotomy injury.

### Knockdown of APC promotes neurite outgrowth *in vitro*

Given previous findings that β-catenin facilitates cellular growth properties, and that it is degraded upon APC complexing, we postulated that APC might act as a regenerative ‘brake’ on neurons. As expected, pre-culture nerve injury (axotomy) significantly increased neurite outgrowth in dissociated DRG sensory neurons (Supplemental Fig. 6). The siRNA protocol was associated with a transfection rate of ~50–65%. Next, we investigated neurite outgrowth and branching in dissociated but uninjured cultured adult primary sensory neurons by knocking down expression using a targeted APC siRNA applied to intact (sham injured) neurons (Supplemental Fig. 7A). Outgrowth was examined after 18–20 h treatment. There was a rise in neurite outgrowth and increased mean total neurite length and branching in neurons with APC knockdown that was dose dependent (Supplemental Fig. 7B).

**Figure 5:**
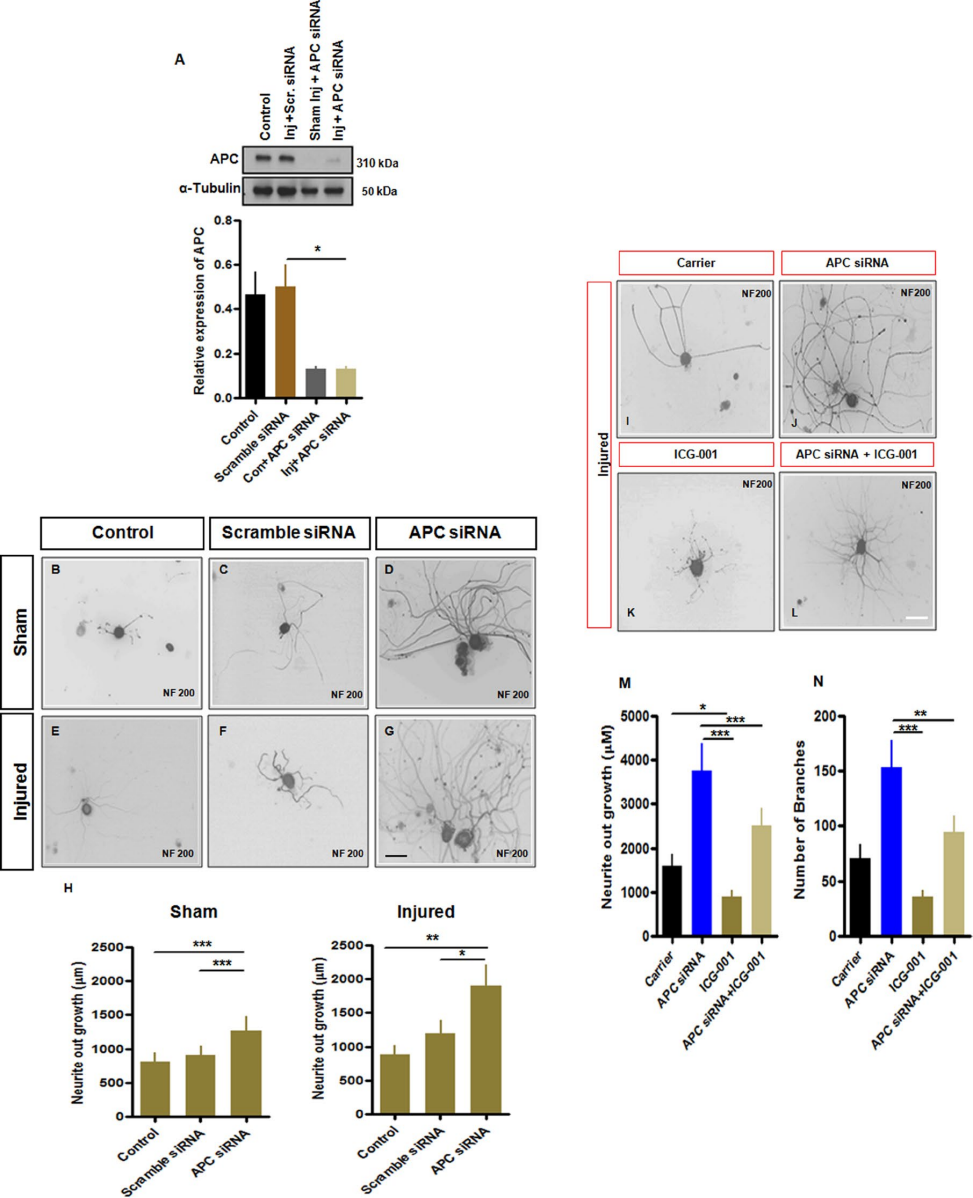
Neurite extension in cultured dissociated rat DRG neurons exposed to APC knockdown *in vitro*. (**A**) Western blot analysis of the expression of APC protein in cultured sensory DRG neuronal cell transfected with siRNA against rat APC (siAPC). (**B**–**D**) The cells were visualized by immunostaining using anti-NF200 antibody of sham injured neurons with or without siRNA: (**B**) sham control, (**C**) with scrambled siRNA and (**D**) with APC siRNA. (**E**–**G**) Representative images of the injured neurons with or without siRNA: (**E**) injured control, (**F**) with scrambled siRNA, (**G**) with APC siRNA. (**H**) Quantification of neurite outgrowth (µm) after outgrowth analysis of cultured DRG neurons of sham and pre-conditionally injured cells. APC knockdown was associated with increased neurite outgrowth in comparison to control cultures after both sham sciatic injury and sciatic axotomy pre-conditioning injury. (**I**–**N**) Pharmacological-mediated knockdown of β-catenin impairs neurite outgrowth in injured cultured primary adult sensory neurons from rats. (**I**) DRG neuronal cells were treated with the carrier (control), (**J**) APC siRNA, (**K**) cells treated within ICG-001 alone, (**L**) cells treated with APC siRNA and ICG-001. ICG-001 was associated with attenuated outgrowth in control and APC siRNA conditions (**M**,**N**). Quantification of total neurite outgrowth of injured cultured neurons. (One-way ANOVA with Turkey post-hoc analysis, *p < 0.05, **p < 0.01, ***p < 0.001, n = 4 separate cultures, means ± SEM, Scale bars are 50 µm).

Next, we examined APC knockdown and neurite extension in control, sham injured and preconditionally injured dissociated primary sensory neurons using APCsiRNA or scrambled sequence siRNA (50nmol). We confirmed 80% knockdown of APC protein in DRG cultures after 18–20 h treatment with APC siRNA when compared to injured scramble sequence siRNA controls (Fig. 5A). Mean total neurite outgrowth in both freshly dissected DRG neurons and preconditionally injured DRG neurons was increased following APC knockdown (Fig. 5B–H). These findings indicated that injured DRG sensory neurons have heightened outgrowth plasticity following knockdown of APC. APC thus attenuated growth in both naive and preconditioned adult sensory neurons. Next, we examined the impact of β-catenin in axotomized DRG sensory neurons on neurite outgrowth. To test this, we studied a specific pharmacological inhibitor of β-catenin signalling. The small molecule inhibitor ICG-001, in DMSO at 10 µM concentration^31,32^ selectively blocks the β-catenin/CBP interaction, thereby interrupting a subset of TCF/β-catenin-mediated transcription activity and also reduced neurite outgrowth in PC-12 cells. In preclinical studies, ICG-001 was sufficient to inhibit tumor progression through attenuation of β-catenin/TCF-LEF signalling^33^. In the present work our control studies used the DMSO carrier (0.1% final). ICG-001-treated cells lowered neurite outgrowth (Fig. 5K) compared with a carrier (Fig. 5I). Whereas APC knockdown, as expected, increased neurite outgrowth (Fig. 5J), combined ICG001 and siRNA APC treatment attenuated this accelerated neurite outgrowth and branch formation (Fig. 5L). These additional data supported the concept, identified above, that axonal branching is impaired when β-catenin action is impaired (Fig. 5M,N).

### Knockdown of APC expression enhances the activity of β-catenin in adult DRG sensory neurons

**Figure 6:**
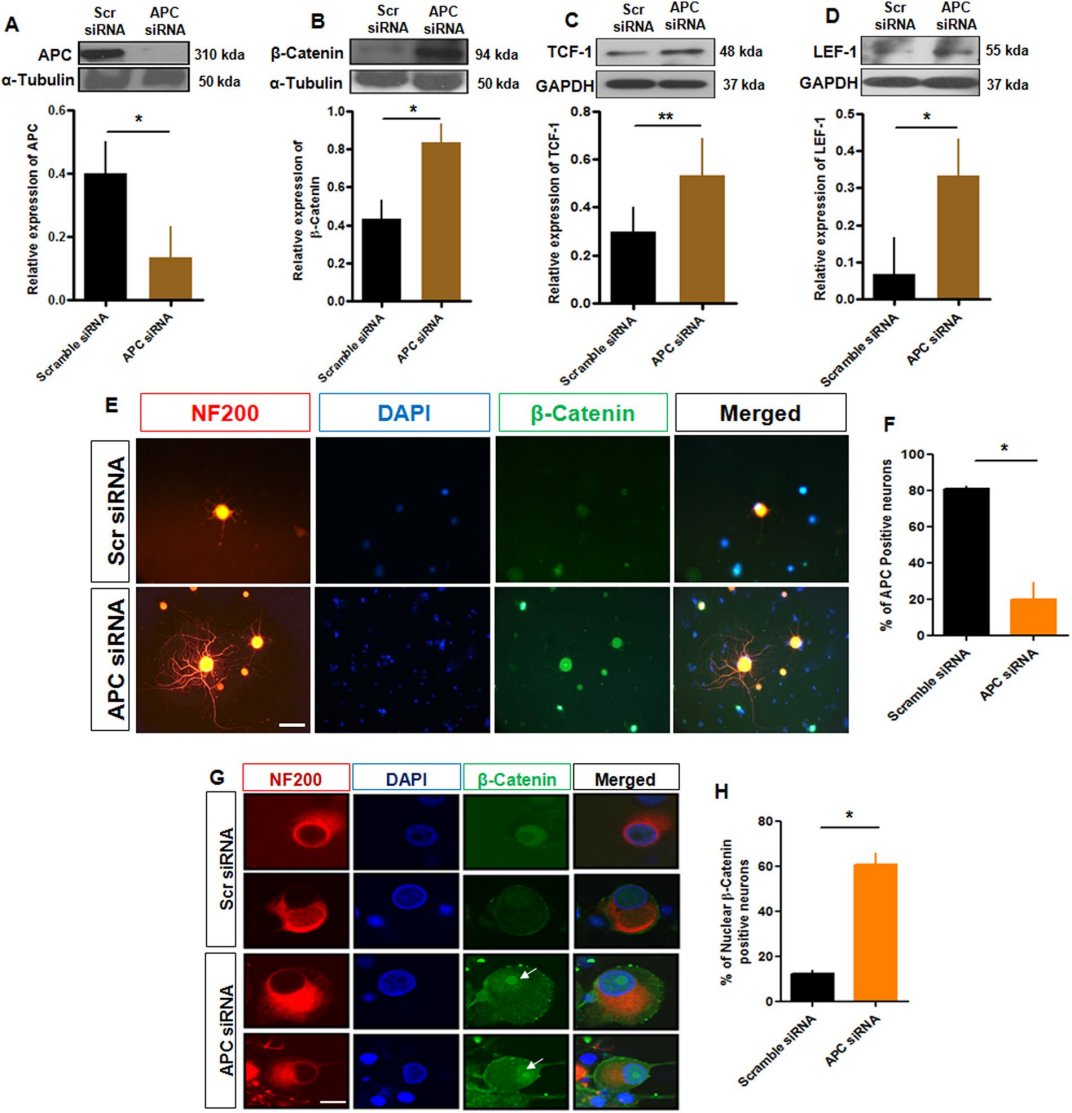
APC regulates β-catenin/TCF/LEF signalling. (**A**–**D**) Cultured DRG neurons were transfected with control siRNA or APC siRNA and then subjected to immunoblotting; protein expression of APC, β-catenin, TCF, LEF and α-tubulin served as an internal control. (**A**–**D**) Quantification of western blot data showing declines in APC and rises in β-catenin, TCF and LEF relative expression. Asterisks indicate a significant difference (*t* test, *p < 0.05; **p < 0.01, n = 3, mean ± SEM). (**E**) Dissociated rat DRG neurons stained for β-catenin, NF200 and DAPI after 18–24 h treatment of siRNA (scrambled or APC siRNA). There was increased expression of β-catenin in the cytoplasm and nuclei in neurons exposed to APC siRNA. Bar = 50 µm. (**F)** Quantification of APC positive neurons in both control and APC siRNA treated condition shows a decreased percentage of APC positive neurons. (**G**) Confocal z stacks demonstrate the cytoplasm and nuclear localization of β-catenin in neurons with APC knockdown when compared to those exposed to scrambled control siRNA. Scale bar = 50 µm. (**H**) Quantification of nuclear β-catenin positive neurons in both control or APC siRNA treated condition shows an increasing percentage of nuclear β-catenin after APC siRNA.

That β-catenin levels are negatively regulated by the APC tumor suppressor protein has been shown in several systems^34^. This reciprocal relationship, involving β-catenin degradation by the ubiquitin-proteasome system^35^ was confirmed above in adult sensory neurons. β-catenin associates with the transcription factors of the lymphoid-enhancing binding factor/T cell factor (LEF/TCF) family to stimulate cell proliferation. To examine this aspect of APC/β-catenin signalling, we further knocked down APC expression in sensory neuronal cells using siRNA (50nmol) as above and examined protein expression. Western blot analysis and immunohistochemistry confirmed APC knockdown (Fig. 6A,F) with reciprocal rises in β-catenin, and TCF/LEF expression (Fig. 6B–D). We further performed immunocytochemistry to map the cellular localization of β-catenin in DRG neuronal cells. In control scrambled siRNA treated cells, β-catenin expression was low level and was distributed in a diffuse cytoplasmic membrane pattern. No nuclear accumulation of β-catenin was present in control neurons (Fig. 6E). In contrast, after APC knockdown, β-catenin in DRG neuronal cells appeared to increase in the cytoplasm, and we observed nuclear localization of β-catenin, confirmed by z plane confocal analysis and quantitation (Fig. 6G,H). Together the findings demonstrate that active β-catenin accumulates in the nucleus upon reduction of APC expression and that this change further stimulates TCF/LEF transcriptional factor expression.

LEF protein expression was higher in sensory neurons in response to ICG001 and siRNA APC treatment which accompanied the attenuated sprouting response compared with ICG001 alone treated cells. This suggested a facilitatory role on Wnt signaling (Supplemental Fig. 8A,B). We validated the pathway further by *in vitro* knockdown of TCF1 using an siRNA in injured neurons (Supplemental Fig. 9A). This approach showed a trend toward reduced neurite outgrowth, albeit not statistically significant (Supplemental Fig. 9B). Together, these results indicate that β-catenin transcription, likely in collaboration with its partners, is a regulator of axon regeneration after peripheral nerve injury.

### APC knockdown improves indices of regeneration following nerve injury

**Figure 7:**
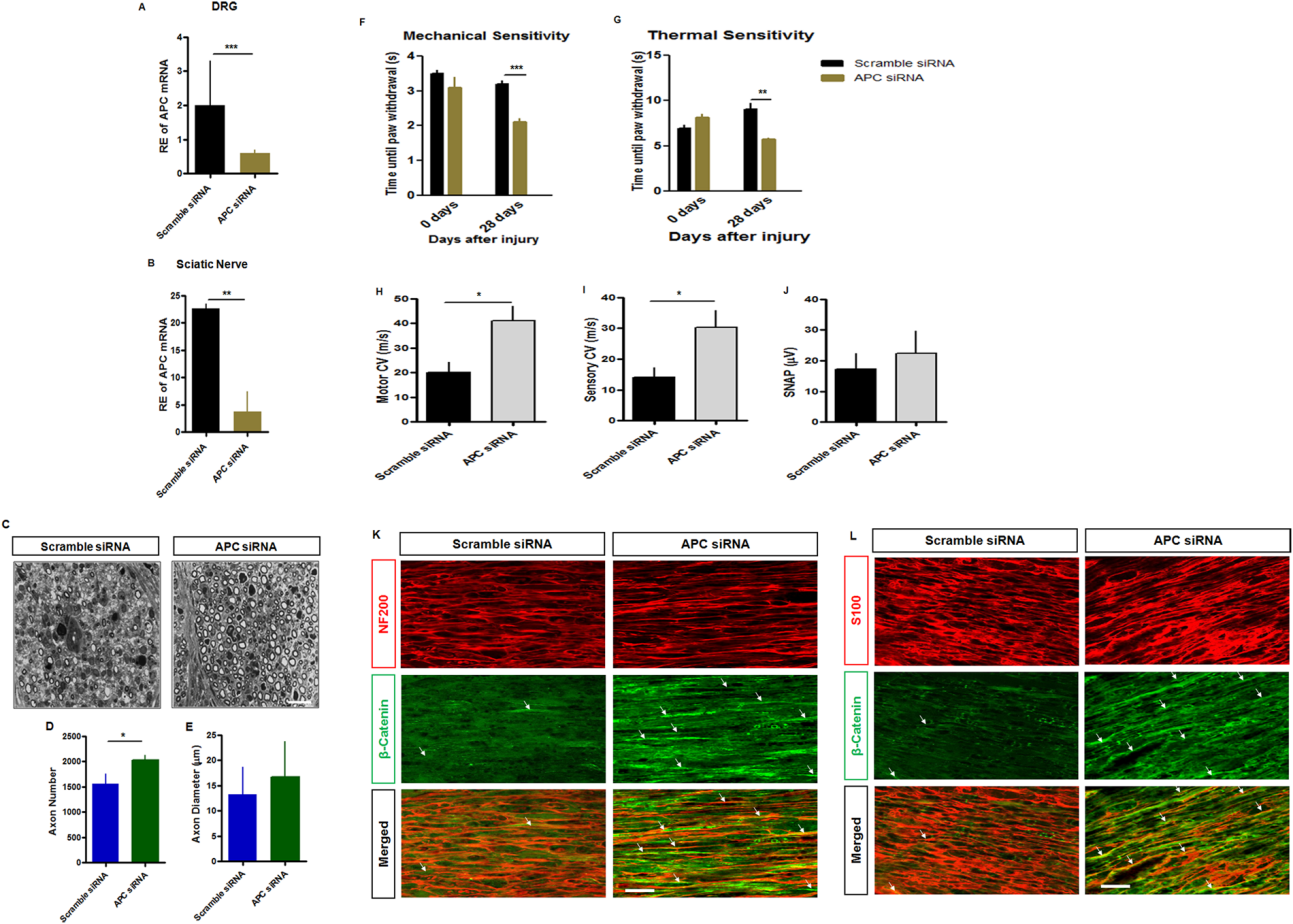
Local knockdown of APC accelerates functional and structural recovery following mouse sciatic nerve crush. (**A**) Knockdown of APC in DRG was confirmed by qRT-PCR. (**B**) Real-time PCR data showing APC knockdown in sciatic nerve. (**C**–**E**) Semi-thin transverse sections of crushed sciatic nerves distal to the zone of injury, after treatment with APC or scrambled control siRNA and analyzed after 28 days. (**D**,**E**) APC siRNA exposed nerves had a greater number of myelinated axon distal to injury indicating more robust recovery of axons, but there was no improvement in axonal caliber. (**F**,**G**) Histograms illustrating recovery of motor and sensory function after peripheral nerve injury [n = 6/group]. There was a rise in mechanical and thermal sensation, indicating recovery by 28 d. (**H**,**I**) A significant impact on the recovery of motor and sensory conduction velocities of regenerating axons at 28d using APC siRNA was noted. (**J**) Sensory nerve action potential amplitudes showed a nonsignificant trend toward higher values in APC siRNA treated when compared to those treated with scrambled siRNA. (**K**) Immunohistochemical analysis of β-catenin distribution in the crushed proximal nerve after treatment with APC or scrambled siRNA. Expression of β-catenin in regenerating axons is indicated using white arrows. Axons labeled with neurofilament (red, NF200) colocalized with β-catenin (green). (**L**) Co-expression of S100 and β-catenin in the crushed proximal nerve after treatment with APC or scramble siRNA. Expression of β-catenin in SC is indicated using white arrows. SCs labelled with s100 (red) are colocalized with β-catenin (green) (*t*-test, *p < 0.05, **p < 0.01, ***p < 0.001, n = 6, means ± SEM, Scale bar = 100 µm).

The impact of APC knockdown on DRG sensory neurons in supporting outgrowth suggested it might offer support of regeneration after nerve injury *in vivo*. Using a mouse model of sciatic nerve crush injury we measured behavioural indices of sensory and motor changes with or without APC knockdown using local administration of siRNA (1.35 µM) applied to the crush site immediately after 15 min, with repeated injections over 5 days post crush injury (Fig. 7). The injection system had the advantage of locally silencing APC in injured axons. We confirmed APC knockdown in the ipsilateral DRG and proximal stump ~ 0.5 cm above the treatment site of sciatic nerve crush area by qRT-PCR (Fig. 7A,B) and immunohistochemistry (Supplemental Fig. 10). Retrograde knockdown in the nerve and parent ganglia has been confirmed using this approach previously in our laboratory^36^. We examined semithin LM transverse sections distal to the site of a sciatic nerve crush injury at 28days (Fig. 7C) to evaluate repopulation of myelinated axons. The number of myelinated axons in APC knocked down nerves was greater in mice treated with APC siRNA. The caliber of the fibers was comparable (Fig. 7D,E). We also evaluated mechanical and thermal sensory recovery and hind paw grip strength (Fig. 7F,G; Supplemental Fig. 11). There (day 0) was no difference pre-injury between both groups in mechanical or thermal sensation. After a sciatic nerve crush injury, APC siRNA treated mice had greater mechanical hyperalgesia, and thermal hyperalgesia. Overall grip strength measures were substantially higher at the 28 day endpoint in both groups after injury compared to pre-injury. However, we noticed that these findings were accounted for by a subset of sporadic high readings during the beginning of the repeated test paradigm, likely secondary to altered positioning of the hindpaw, secondary to injury, during testing. Eliminating these isolated readings eliminated this apparent rise and provided readings comparable to the uninjured mice (Supplemental Fig. 11). With this caveat, there was a small improvement in grip strength power in the APC siRNA treated group (both with the sporadic high measures included or eliminated). Local administration of APC siRNA at the injury site was associated with improved motor and sensory conduction velocities of regenerating myelinated axons (Fig. 7H–J). There was a nonsignificant trend toward higher amplitude sensory nerve action potentials (SNAPs) compared with the scrambled siRNA control groups. In addition, the proximal nerve processed for β-catenin immunohistochemistry following APC siRNA exposure appeared to express higher levels of β-catenin in regenerating axons and SC although this was not quantitated (Fig. 7K,L).Overall our findings provided behavioral, electrophysiological and structural evidence of an impact on nerve regeneration associated with APC knockdown. The results support the hypothesis that β-catenin, enabled by APC knockdown, plays an important role in axon regeneration following nerve injury *in vivo*. These ideas are captured in a simplified summary of the potential role of the β-catenin/TCF/LEF signalling pathway in peripheral neuron regeneration (Supplemental Fig. 12).

## Discussion

The major findings of this work were (i) primary sensory neurons and axons express the reciprocal signaling partners APC and β-catenin; APC rises and β-catenin declines following axotomy injury; (ii) knockdown of APC in adult sensory neurons increases their neurite outgrowth and increases β-catenin nuclear expression; (iii) rises β-catenin and LEF-TCF expression are associated with increased neurite outgrowth following nerve injury in the setting of APC knockdown; inhibition of β-catenin activity blocks enhanced APC siRNA related outgrowth *in vitro*. (iv) *In vivo*, local knockdown of APC, at the site of a sciatic nerve crush injury is associated with indices of improved functional and structural recovery. Overall, the findings support important roles for reciprocal and meaningful APC-β-catenin signalling during injury and recovery following peripheral nervous system injury.

While peripheral neurons exhibit some capacity to regenerate, in contrast to severe limitations in the CNS, the extent of recovery remains limited and the timetable is prolonged. It is a misconception that peripheral nerve injuries are capable of robust or full regeneration: functional deficits following major nerve trunk injuries or axonal disease remain severe and persistent. Given these issues, there is significant interest in unraveling a range of molecules and pathways that impact the growth properties of postmitotic neurons. Transcription factors can activate or repress target gene expression by binding multiple promoter regions with diverse impacts on a large number of genes. Inducing the expression or activity of transcription factors and coordinately blocking the function of transcriptional repressors provides an efficient way to enhance the regenerative potential of neurons^37,38^.

In this work, we addressed the role of the transcriptional repressor APC, a tumour suppressor molecule, and its partner β-catenin, during axon regeneration following peripheral nerve injury. We identified a role for this canonical pathway as regulated by APC. β-catenin-TCF/LEF transcriptional factors are essential in neuronal fate specification^11^, dendritogenesis, synapse formation, neurite outgrowth, neural regeneration, and cell survival through prevention of apoptosis^9,39^. Previous reports showed that the role of the Wnt/β-catenin pathway in neurite outgrowth has been examined in other neuronal or neuronal-like cells^5,40^. β-catenin inhibition with small molecules in PC 12 cells deregulates β-catenin/TCF/LEF signalling and inhibits neuronal differentiation and outgrowth^32^. These interactions are complex however. For example, APC deficient cortical neurons exhibit exuberant axon branching *in vitro*^41^. APC has been shown to be involved in cell cycle control of neuronal-like PC12 cells, and apoptosis of neural crest cells^22,23^. In our work, we identify paradoxical rises of APC protein in DRG after injury, when enhanced growth is advantageous. Moreover, the primary sensory neurons expressing APC were of a variety of cell sizes but there was particularly intense expression involving small caliber neurons that colocalized with IB4-expression. Usually, IB4 small positive neurons have lower levels of the expression of the heavy neurofilament subunit in transverse section of DRG ganglia. *In vitro* this distinction is usually less prominent when the neurons are dissociated. IB4-binding-positive neurons are nociceptive, innervate the epidermis and demonstrate attenuated growth properties both *in vitro* and *in vivo*. Following nerve injury, the epidermal terminals of IB4 neurons retract, a finding potentially linked to skin hyperalgesia. Our previous studies^30^ have shown that sensory neurons expressing glial cell-derived neurotrophic factor coreceptor α1 (GFRα1), which is largely overlapping with IB-4 neurons, had lesser baseline growth and plasticity. Similarly, Tucker *et al*.^42^ showed that IB-4 neurons have impaired neurite growth, although they are capable of growth after exposure to GDNF and laminin. Our results are in line with these findings and suggest that APC is prominently (although not exclusively) expressed in IB4-positive neurons after nerve injury and may serve as a mechanism of neurite growth inhibition. It is interesting that this population also expresses a higher level of PTEN, an additional inhibitor of growth^1^. Why two growth ‘brakes’ should be similarly upregulated in this population of neurons is interesting and not known at this time. Notably, the injury-related upregulation of APC was also expressed in Schwann cells and in axons regenerating in the proximal stump of the injury site. Our results are consistent with upregulation of APC proteins and downregulation of β-catenin protein *in vivo*, which may promote apoptosis through enhancement of the β-catenin destruction complex involving proteasomal degradation of ubiquitinated proteins^19,39^.

In contrast, the Wnt/β-catenin signal is activated in tumour cells when APC is non-functional. This drives high levels of β-catenin accumulation in the cytoplasm that translocate into the nucleus, form a complex with TCF/LEF factors and activate Wnt target genes involved in cell survival, proliferation, and differentiation. Activated Wnt signalling plays a central role in intrinsic regeneration in the adult retina, and activation of the signalling promotes retinal neuron regeneration after injury. Lie *et al*.^43^ have also shown that Wnt/β-catenin signalling is required for adult hippocampal neurogenesis under physiological conditions. In post mitotic neurons, overexpression of β-catenin together with cadherins, participates in dendritogenesis, synaptogenesis and synaptic formation^44^. There is also a role for Wnt/β-catenin signalling in regulating synaptic plasticity and axonal growth. Similarly, the N-and C-terminal domains of β-catenin enhancement mediate the cell-cell adhesion or promote axon branching in CNS neurons *in vivo*^45^. In our work, APC siRNA enhanced levels of intracellular β-catenin that accumulated in the cytoplasm and nucleus where it is expected to form a complex with T-cell factor or lymphoid enhancer factor (LEF/TCF) proteins; this was associated with robust increases in neurite outgrowth. It is remarkable that the impact of APC was substantially greater in preconditioned neurons, indicating persistent expression of this block to regrowth previously considered optimal for neurons. However, pharmacological inhibition of β-catenin blocked the impact of APC knockdown. In separate work, blocking Wnt/β-catenin signalling was shown to abolish neurogenesis in adult hippocampal progenitor cells *in vitro* and suppress neurogenesis *in vivo*^43^. Notably, β-catenin disruption also showed increased neuronal cell death, suggesting that the Wnt/β-catenin/LEF pathway controls not only the generation but also the survival of newly generated neurons^46^.

Here we also note that siRNA-mediated APC protein suppression *in vivo* improved indices of peripheral nerve regeneration following sciatic nerve crush. The findings may differ from those of Saijilafu *et al*.^28^ who did not identify an impact of β-catenin knockdown on regrowth of sensory axons after a nerve crush in mice. This paper identified key roles for PI3K in supporting peripheral nerve regeneration, operating through activation of pAkt, inactivation of GSK3 and induction of Smad1. Their approach, involved DRG electroporation and siRNA knockdown of β-catenin 2 days before crush and 5 days before harvesting and evaluation. It is possible that early knockdown within DRGs may be transient and if carried out early, might not achieve persistent knockdown during early regrowth. Our approach was to repeatedly apply siRNA through the first week of regenerative regrowth when it is possible that β-catenin expression is more important.

While not shown here, crush injury characteristically is associated with an earlier (approximately 14 days) decline in the sensitivity of the hind paw to thermal and mechanical sensation. While this usually improves somewhat by 28d, in the present work, limbs exposed to local APC knockdown had a remarkable gain in the sensitivity of the limb to both sensory modalities. In the case of mechanical sensitivity, it is possible that our results parallel findings of allodynia identified as mediated by β-catenin^18,19^. Despite this caveat we did identify evidence of enhanced regeneration in our model after APC knockdown. Regenerating axons had evidence of greater maturity with higher conduction velocities. The numbers of myelinated axons distal to the injury zone were substantially greater in APC knockdown nerves. While the findings suggested a direct neuronal and axonal impact, we cannot exclude impacts on local Schwann cells and myelination independently. While conduction velocities were higher, mean axon calibers were similar, but this likely reflects repopulation by added numbers of smaller caliber regenerating axons not captured by the conduction studies (which emphasize the fastest conducting fibers). As in previous work, a non-viral siRNA approach applied locally to a site of axon damage provided knockdown of the inhibitory protein locally and in the ipsilateral DRG. Additional work *in vivo* could include longer term studies such as assessment of skin reinnervation.

In summary, the present study provides evidence that APC/β-catenin signalling pathway impacts the regeneration of neurons after injury in the adult mammalian peripheral nervous system. Manipulation of this pathway influences neuronal plasticity *in vitro* and nerve regrowth *in vivo*. As in other systems, this canonical signaling pathway appears to be a key player in neuronal regrowth.

## Methods

### Animals

Adult male Sprague–Dawley rats (180–200 g) and CD-1 mice (18–20 g) were obtained from Charles River Laboratories, Quebec, Canada. All animals were housed on 12-h light/dark cycle with food and water *ad libitum*. Animal care and all experimental protocols were reviewed and approved by the University of Calgary and University of Alberta Animal Care Committees and followed the guidelines of the Canadian Council of Animal Care guidelines. Experiments were designed to minimize the number of animals required and to minimize animal suffering as per Canadian Council on Animal Care guidelines.

### Surgery

Before surgery, rats or mice were anesthetized with Isofluorane (Abbot Laboratories, Saint-Laurent, Canada). Under sterile conditions, the left sciatic nerve was cut or crushed at the mid thigh level, the skin wound was sutured with 3–0 silk suture and the rats or mice were caged individually for three days. For sham injury, the left-side skin was cut at the mid thigh level, and the muscle was displaced to expose the sciatic nerve, but the nerve was not injured. Buprenorphine jello was provided once a day after the injury for analgesia.

### Culture of primary dorsal root ganglia (DRG) neurons

Before tissue harvesting, rats were anesthetized with Isoflurane (Abbot Laboratories) and then killed three days following the conditioning lesion or sham surgery. Primary adult DRG neurons were dissociated and maintained *in vitro* using a modification from the method of Lindsay^47^. Briefly, 35 mm dishes were coated with 0.1% poly-L-lysine (Sigma, St Louis, MO, USA) overnight and next morning washed 3 times with double distilled water and coated with 0.01% laminin (Sigma) for 1 h at 37 °C. L4-L6 DRGs were removed from the rats and placed into L15 medium (Invitrogen), where the axon roots and dural tissue were manually removed. The DRG were washed three times in cold L15, then DRGs were incubated for 1.5 hours at 37 °C in L15 containing 1 mg of collagenase per ml. After multiple pipetting, the solution was centrifuged at 800 rpm for 5 minutes, followed by three washes in L15. Cells were resuspended in 1 ml of L15, and passed through a 70 micron mesh. Then the cell solution was loaded gently onto 2 ml of 15% BSA in L15 and centrifuged at 900 rpm for 10 minutes. The pellet cells were washed once in L15, and resuspended in DMEM-F12 (Invitrogen) medium containing 1% N2 supplement (Invitrogen), 10 µM cytosine β-D-arabinofuranoside hydrochloride (Sigma), 0.5–0.8% BSA, 0.2 ng/ml NGF (Cedarlane Laboratories) and 1% penicillin/streptomycin. Finally, cells were plated immediately on 4-well plates, previously coated with poly-L-lysine. The plate was maintained in an incubator at 37 °C with 5% CO_2_. For *in vitro* inhibitor studies, neuronal cultures were incubated with 12 µM concentrations of the β-catenin pharmacological inhibitor (ICG-001), were treated with culture medium, and the carrier DMSO treated cells served as control. In separate experiments APC siRNA (Qiagen), or scrambled siRNA were transfected using HiPerFect Transfection reagent (Qiagen) according to manufacturer’s instructions. Briefly, per well of a 4-well plate, 5 µl transfection reagent and 50nmol siRNA (2.5 µl) were each added to 50 µl DMEM without supplements. The two solutions were combined and the complexing reaction was allowed to continue for 20 min at room temperature. One hundred microlitres of the siRNA-transfection reagent complex were added to each well. Cultures were kept for 18–20 h then fixed and processed for immunocytochemistry.

### Immunohistochemistry on frozen sections

Tissue samples from rats were fixed in modified Zamboni’s fixative (2% paraformaldehyde (PFA), 0.5% picric acid, and 0.1% phosphate buffer) overnight at 4 °C. Tissues were then washed in PBS three times, cryoprotected in 20% sucrose/PBS, and left at 4 °C overnight. After embedding in optimum cutting temperature (OCT) compound (Miles), 14-μm-thick sections were placed onto poly-L-lysine-coated slides. The slides were probed for APC (Sigma, 1:50), β-Catenin (Sigma, 1:100), and double labelled with anti-NF-200 (Sigma, 1:400), anti-S100 (1:200, Sigma) or unconjugated IB4 lectin (Vector Laboratories) followed by goat anti-IB4 (Vector Laboratories, 1:8000), and incubated at 4 °C for 24 h then rinsed three times in PBS. Secondary antibodies were anti-mouse IgG CY3 conjugate (1:100, Sigma) and Alexa Fluor 488 goat anti-rabbit IgG conjugated (1:100, Sigma) for 1 h at room temperature. Finally, cover slips were mounted on the slides with bicarbonate buffered glycerol (pH 8.6) and the slides were viewed with a multiphoton microscope (Nikon A1R). Negative controls included omission of primary antibodies on parallel sections (Supplemental Fig. 13). We validated the expression of APC in IB4 positive neurons as follows: (+) when the staining was mildly intense; moderate (++) when between one-third and two thirds of a neuron stained moderately; and strong (+++) when the majority of the neuron (> two-thirds) stained intensely. Within the DRG after injury, a majority of IB4 positive neurons showed strong expression (> two-thirds) of APC staining when compared to control (+) IB4 positive neurons (Fig. 2I). In some experiments, slides were counted and analyzed for the average number of positively stained cells. The results were expressed as a % of total cells in six randomly selected fields.

### Immunocytochemistry

Cultured DRG neurons from rats were fixed with 4% warmed paraformaldehyde for 15 minutes followed by PBS, given twice for 15 minutes each. Next, the dishes were blocked with 10% goat serum (0.3% Triton X100/PBS) for 30 minutes at RT. Neurons and neurites were stained with 1-hour incubation of primary antibodies including mouse anti-neurofilament 200 (NF200) (1:800, Sigma Aldrich), rabbit anti-APC (1:50, Santa Cruz) and rabbit anti-β-catenin (1:50, Sigma), incubated overnight at 4 °C. After washing with PBS twice for 15 minutes each, secondary antibodies [CY3 sheep anti-mouse (1:500, Sigma Aldrich) and Alexa Fluor 488 goat anti-rabbit (1:500, Invitrogen)] were applied following PBS at room temperature for 1 h. The samples were then washed in PBS and mounted using DAPI vectashield (Vector laboratories) to label the nuclei and imaged using a Zeiss Axioskope fluorescent microscope with digital camera and Axiovision imaging software (Axioskope, Axiovision and Axiocam, Zeiss Canada). Adobe Photoshop software was used to merge images. For neurite extension assays, the NF200-labeled DRG neurons was photographed and the pictures were analyzed by MetaExpress software to measure the total neurite growth, the number of neurites per cell body, and the mean cell body size based on fluorescent intensity (of NF200) above background; this is set by program parameters based on the level of background. Number of primary branches were quantified by the MetaExpress program (Molecular Devices, Sunnyvale, CA). For analysis between 40 and 60 neurons were analysed per condition and per culture day (3 culture day, 180 neurons total for each condition).

### Quantitative reverse transcription polymerase chain reaction (qRT-PCR)

Quantitative reverse transcription-polymerase chain reaction was performed according to previous descriptions^1^. Briefly, total RNA samples from non-injured and injured sciatic nerve tissue from mice was extracted using an TRIzol Reagent (Invitrogen, Burlington, Canada) according to the manufacturer’s instructions One μg of total RNA was treated with DNAse (Promega, Madison, WI) and processed to complementary DNA (cDNA) synthesis using the Super Script II Reverse Transcriptase (Invitrogen). Primers used were as follows: APC1 F 5′-TGACCTTTGGAGATGTTGCCA-3′; APC1, R 5′-CCGCAAAACACTTGCAATAACC-3′ RPLPO: F, 5′-AAGAACACCATGATGCGCAAG-3′; R, 5′-TTGGTGAACACGAAGCCCA-3′.

Products were labelled using SybrGreen I fluorophore (Invitrogen). The cycle number at which the fluorescence signal crossed a fixed threshold (threshold cycle *C*_T_) with an exponential growth of polymerase chain reaction product during the linear phase was recorded. Relative expression values were generated using the comparative *C*_T_ method (2^−∆∆*C*T^), where all genes of interest were standardized to the expression of RPLPO.

### Western immunoblot analysis

Sciatic nerve and DRG (L4-L5) samples were prepared in RIPA lysis buffer (Sigma), containing a protease inhibitor cocktail. Proteins were separated by 10% sodium dodecyl sulfate-polyacrylamide gel electrophoresis. Separated proteins were transferred onto PVDF membrane in Tris-glycine-methanol buffer for 2 h at 4 °C. Membranes were blocked for 1 h at 25 °C in phosphate-buffered saline with 0.1% Tween 20 supplemented with 5% non-fat milk. They were then incubated overnight with the respective antibodies: APC (1:200, anti-rabbit, Santa Cruz Biotechnology), β-Catenin (1:2000, anti-rabbit, Sigma), LEF (1:500, anti-goat, Santa Cruz Biotechnology Inc.), TCF (1:500, anti-goat, Santa Cruz Biotechnology Inc.) and tubulin or actin (1:2000, mouse monoclonal, Sigma) as a loading control in 2% bovine serum albumin in Tris-buffered saline (TBS). The membranes were then incubated for 1 h at 37 °C with a goat anti-rabbit and a goat anti-mouse immunoglobin antibodies conjugated to horseradish peroxidase and diluted, respectively, at 1:10,000 and 1:10,000 in TBST containing 0.1% Tween 20 and 5% non-fat milk. Immunoreacting bands were detected using the enhanced chemiluminescence (ECL) detection reagents. Quantification of bands was through Adobe Photoshop and the band densities were normalized with those of the loading control.

### *In vivo* siRNA application and functional recovery

The control (scramble) siRNA or APC siRNA were transfected using HiPerfect Transfection reagent (Qiagen) according to manufacturer’s protocol. For *in vivo* injection, 4.05 µl of siRNA (1.35 µM) were mixed with 20 µl HiPerfect Transfection reagent and 35.95 µl saline. An injection of 40 µl of siRNA preparation was administered to each mice crush site, and remaining 20 µl transdermally into the hind paw on the crush side for 7days.

The experimental mice underwent mechanical (Von Frey filaments), hindpaw grip strength and thermal testing at Day 0 (before crush injury), and 28 days following sciatic nerve crush, There were 5-min intervals provided between a total of 3 trials performed during the same day. Mechanical sensitivity was measured using an automated Von Frey apparatus (dynamic plantar anaesthesiometer, UGO). A filament with a progressively-increasing force (2 g/s) was applied to the plantar surface of the mice through the wired mesh until a withdrawal reflex was noted. Three separate trials were conducted and the mean latency (time to withdraw) and amount of threshold force were calculated. For testing the recovery of thermal sensation, we used the Hargreaves apparatus^48^. In brief, a radiant heat source was applied individually to the middle of the hindpaw and the latency (in seconds) to withdrawal was measured. Three separate trials were performed for the withdrawal response. Mechanical and thermal testing was performed on identical days with an interval of at least 1 h between the two tests. Analyses were done with the observer blinded to the nature of the treatment group.

### Electrophysiological Evaluation

Motor and sensory electrophysiology were carried out under isoflurane anesthesia, as previously described^49^. Motor sciatic nerve conduction recordings were carried out using stimulation at the sciatic notch and knee with recording over interosseous foot muscles in mice. Sensory sciatic nerve conduction recordings were carried out by stimulation of the toe digital nerves with recording at the knee. Near-nerve temperatures were maintained at 37 °C ± 0.5 °C.

### Semi-thin Sections

Ipsilateral, sciatic nerves distal (~10 mm) to the crush site were harvested at 28 days after sciatic crush. Mice underwent sciatic crush then daily application, for 6 days, of APC or scrambled siRNA. Sciatic nerves were fixed in glutaraldehyde (2.5%) buffered in cacodylate (0.025 M) overnight, washed, and stored in cacodylate buffer (0.15 M), then post-fixed in osmium tetroxide (2%), washed in graded alcohols then embedded^50^. Transverse sections at 1-µm thickness were made through the approximate center of the crush area and stained with toluidine blue for light microscope morphometry.

### Analysis

Statistical analysis using one-way ANOVA with Tukey post‐hoc analysis was performed for neurite extension data. When only two experimental groups were compared, a Student’s t-test was used.

## Electronic supplementary material

Supplementary Information
