# Supplementary material for: Expression and Manipulation of the APC-β-Catenin Pathway During Peripheral Neuron Regeneration
Source: Sci Rep. 2018 Sep 4;8:13197. doi: 10.1038/s41598-018-31167-1 (PMC6123411; doi:10.1038/s41598-018-31167-1)

EXPRESSION AND MANIPULATION OF THE APC-β-CATENIN PATHWAY DURING PERIPHERAL NEURON REGENERATION

Arul Duraikannu[1, 2], Jose Martinez[2], Ambika Chandrasekhar[1] and Douglas W. Zochodne[1, 2]*

[1]Division of Neurology, Department of Medicine,& Neuroscience and Mental Health Institute, University of Alberta, Edmonton, Canada
[2]formerly of Hotchkiss Brain Institute and Department of Clinical Neurosciences, University of Calgary, Calgary, Alberta

Supplemental Figures

Supplemental Figure 1: Immunohistochemical analysis of APC expression in the lumbar spinal neurons of control and injured rats. A representative image of spinal motor neurons shows the presence of APC in naive and sciatic nerve-injured animals. Scale bar=50µm**.**

Supplemental Figure 2: (A-H) Immunofluorescent staining in dissociated DRG neurons demonstrates the expression of APC in both sham and pre-conditionally injured neurons, using the indicated antibodies; NF200 (red), APC (green), DAPI (blue). Scale bars=50µm. At a higher magnification, shown as insets, there was ubiquitous expression of APC involving cytoplasm, nucleus and their axons of DRG neurons after injury. I, Western blot indicates the expression of APC (310 kDA) and α-tubulin (50 kDA) in control and 3 day injured DRG cultures showing that APC expression is increased after rat sciatic nerve axotomy. (*t*-test, ***$p<0.001$, $n=3$, means±SEM).

Supplemental Figure 3: Subpopulations of IB4 and NF200 labelled neurons in DRG cultures. The pie chart indicates the percentage of subpopulation expression of IB4 (37%) and NF200 (63%) labeled neurons in DRG dissociated cultures (*in vitro*). Note, that IB4 neurons did have some degree of NF200 costaining, albeit not as intense as the IB4 expressing neurons. Higher expressing NF200 neurons (63%) did not have costaining with IB4.

Supplemental Figure 4: Immunohistochemical analysis of β-catenin and colocalization in intact uninjured IB4 DRG neurons. Representative images show a control DRG, using the indicated antibodies; IB4 (Green), β-catenin (red) and DAPI (Blue). β-catenin expression included that within small nonpeptidergic neurons. Scale bar=50µm

Supplemental Figure 5: Previous sciatic axotomy nerve injury diminished β-catenin expression within parent neurons *in vitro*. (A-H) Immunofluorescent staining in dissociated rat DRG neurons demonstrated the expression of β-catenin with neurons using anti-NF200 and β-catentin antibody of sham-injured and pre-conditionally injured

cultured neurons. β-catenin is expressed in cultured DRG neurons in both sham and pre-conditionally injured neurons, using the indicated antibodies; NF200 (Red), β-catenin (Green) and counter stained with DAPI for nuclei (blue) Scale bar=50μm. I, Western blot show the expression of β-catenin (94 kDA) and α-tubulin (50 kDA) in control and 3 day injured DRG cultures showing that β-catenin expression is decreased after rat sciatic nerve axotomy. (*t*-test, *$p<0.05$, n=3, means±SEM).

Supplemental Figure 6: Analysis of neurite outgrowth in sham-injured and preconditioned-injured neurons *in vitro*. Quantification indicates there was significant difference between the two groups. (*t* test,*$p<0.05$ n=4, mean±SEM).

Supplemental Figure 7: Varying concentrations of APC siRNA tested on cultured primary sensory neurons outgrowth *in vitro* applied to sham injured/pre-conditioned injured neurons from rats and labeled with NF200. (A) Representative images demonstrating that APC siRNA treatment is associated with a dose dependent rise in neurite outgrowth up to 50 nM. Scale bar=50μm. (B) Quantitative analysis of neurite outgrowth in sham-injured neurons (One-way ANOVA with Turkey post-hoc analysis, ***$p<0.01$, n=3 separate cultures, mean±SEM, scale bars are 50μm).

Supplemental Figure 8: LEF-1 expression is elevated in pharmacological-mediated inhibition of β-catenin and APCsiRNA treated cultured primary adult sensory neurons from rats. (A) DRG neuronal cells were treated with ICG-001 alone and APC siRNA/ICG-001. (B) Quantification of protein data from A. Note that LEF-1 expression is significantly increased in APCsiRNA and ICG-001 associated treated neurons. (*t*-test, ***$p<0.001$, n=3, mean± SEM).ICG-001 mediated knockdown of β-catenin in DRG neurons partially prevents the LEF synthesis after APC knockdown.

Supplemental Figure 9: Impact of TCF siRNA knockdown on TCF protein and neurite outgrowth *in vitro* applied to sham injured/pre-conditioned injured cultured primary adult mice neurons. (A) Western blot analysis of the expression of TCF protein in cultured sensory DRG neuronal cell transfected with siRNA against TCF (siTCF). (B)

Quantification total neurite outgrowth of injured cultured neurons shows a trend, although not statistically significant toward reduced neurite outgrowth (One-way ANOVA with Turkey post-hoc analysis, *$p<0.05$, n=3 separate cultures, mean±SEM).

Supplemental Figure 10: Local knockdown of APC in sciatic nerve was confirmed by immunohistochemistry. Expression of APC in the crushed proximal nerve after treatment with APC or scramble siRNA, using the indicated antibodies; APC (Green), and NF200 (red). APC was expressed on very few of the neurofilament positive axons in the APC siRNA exposed nerves when compared those treated with scramble siRNA. Scale bar=100µm.

Supplemental Figure 11: Summary of hind paw grip strength assessments. Silencing APC following sciatic crush mice by siRNA improves grip strength recovery. Three repeated test trials were conducted. Note that initial trials of injured paws had elevated values that returned to comparable ranges with subsequent trials. This (elevated Trial 1 values) is assumed to be an artefact of testing post injury. The values of test generated between groups are comparable at 28 days. (*t*-test, *$p<0.05$, **$p<0.01$, n=6,means±SEM).

Supplemental Figure 12: A simplified scheme of the hypothesized β-catenin-LEF/TCF signal pathway in PNS regeneration. There is normally interaction of APC with AXIN and GSK3 that phosphorylates β-catenin to be rapidly degraded by the ubiquitin proteasome. In the absence or knockdown of APC signal with an altered AXIN- GSK3β complex, β-catenin accumulates in the cytoplasm and nucleus and forms a complex with lymphoid enhancer factor/T cell factor (LEF/TCF) in the nucleus, which initiates transcription of downstream target genes and promotes regeneration.

Supplemental Figure 13: Fluorescence control analysis of immunoreactive sections. Negative controls were processed in parallel in all experiments, with primary antibodies omitted. Nonspecific signals were not detected from DRG, sciatic nerve and neurons. Scale bar=100µm.

Supplemental Figure 1.

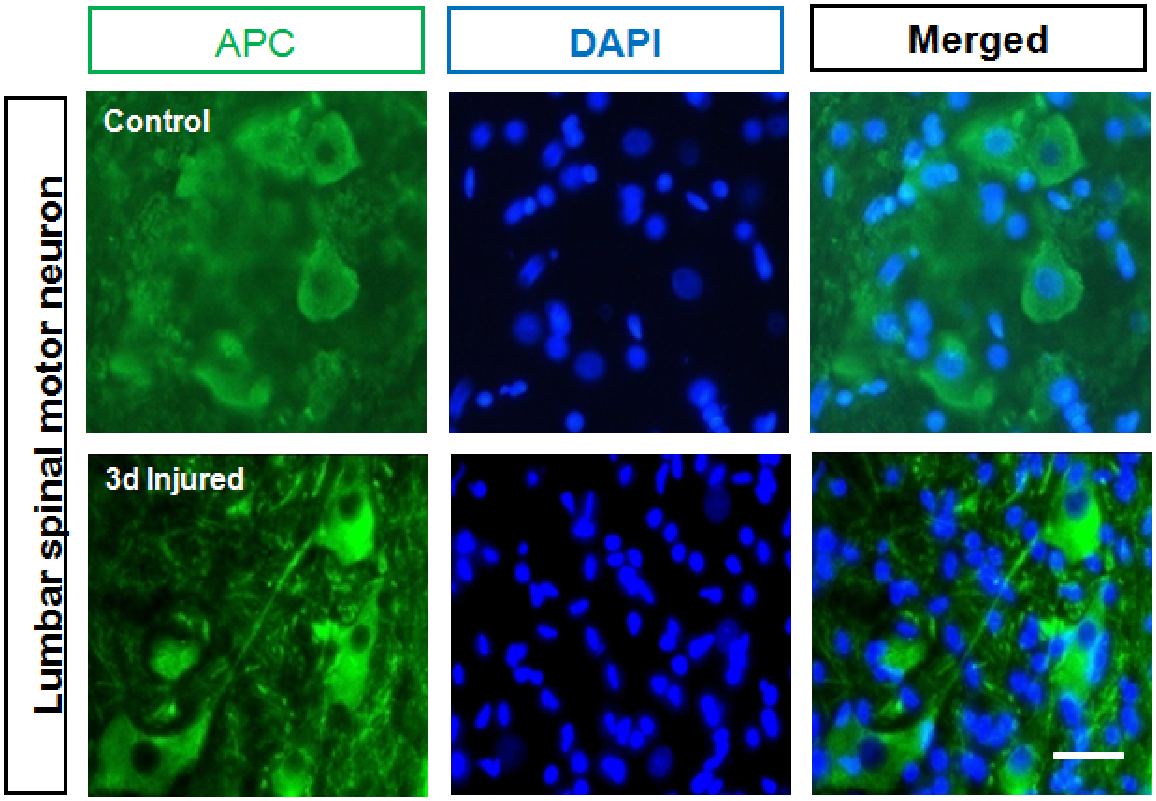

Supplemental Figure 2.

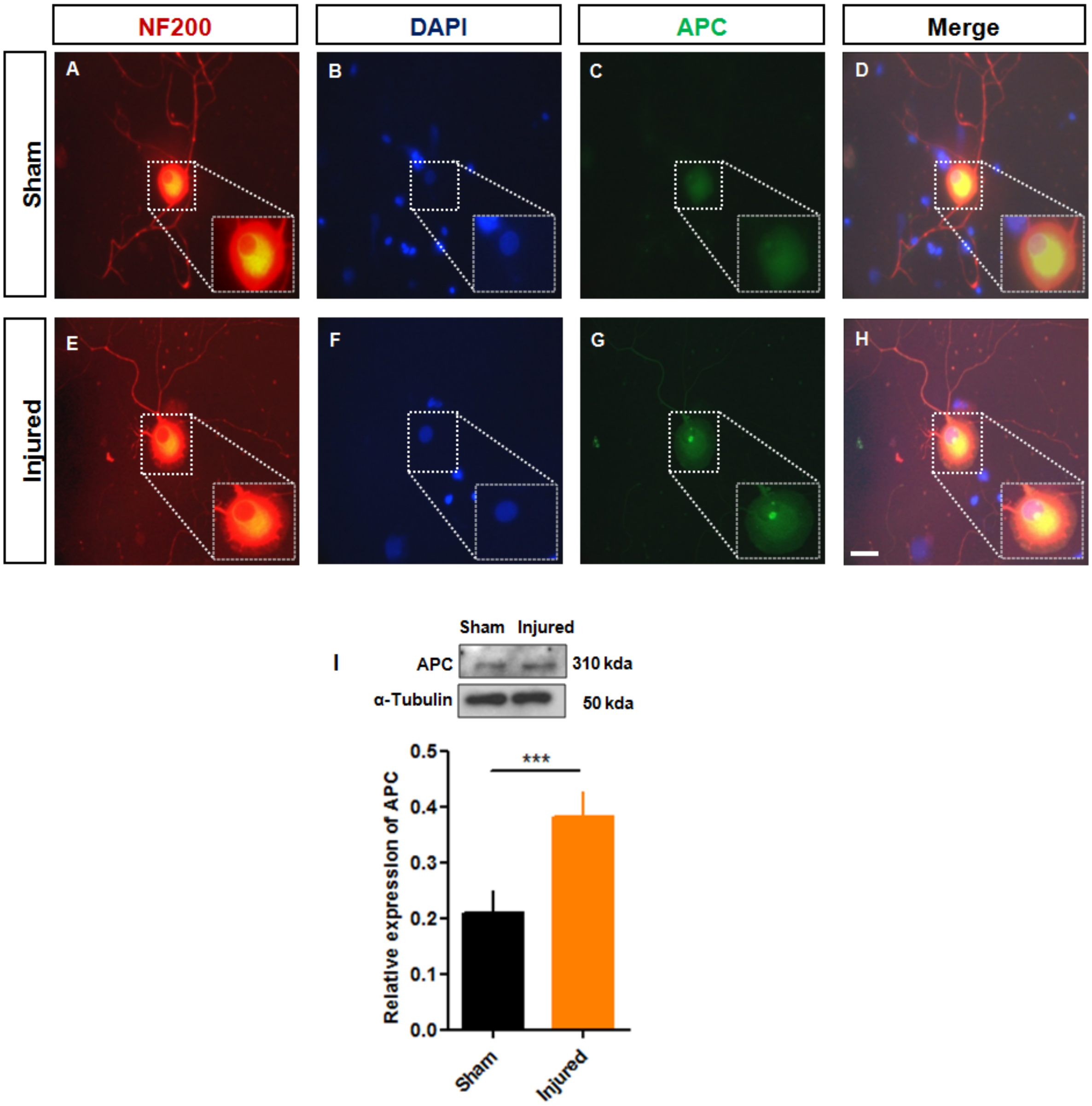

Supplemental Figure 3.

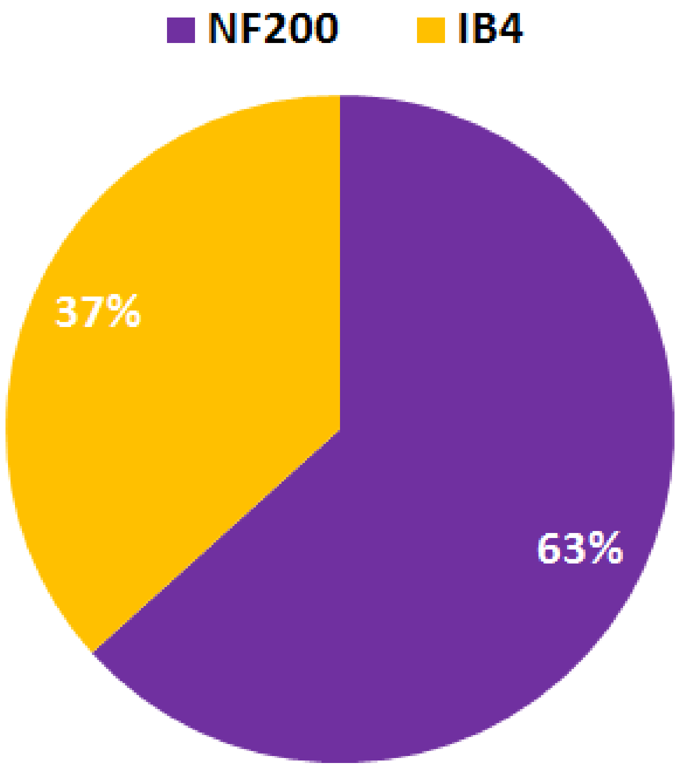

Supplemental Figure 4.

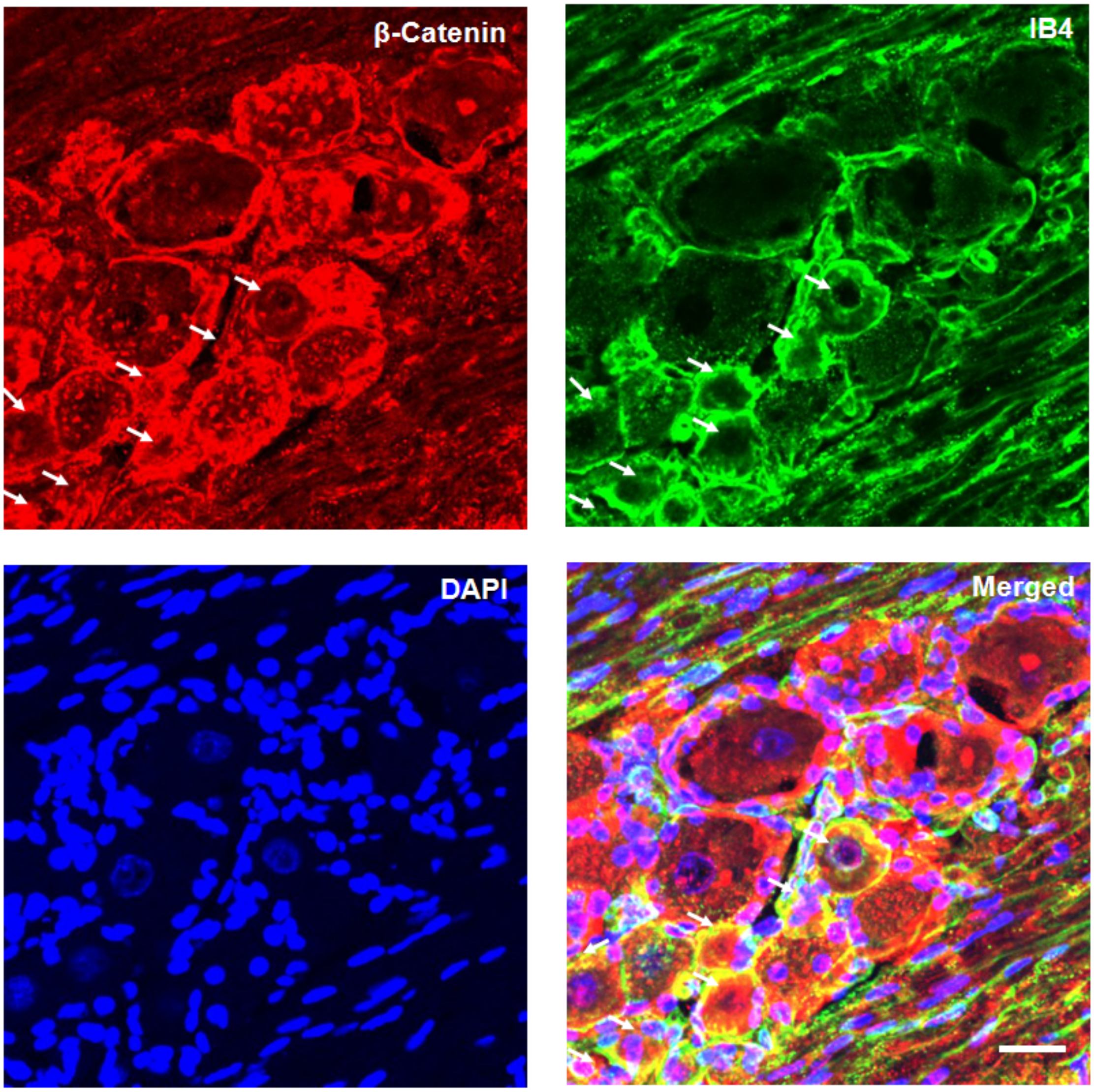

Supplemental Figure 5.

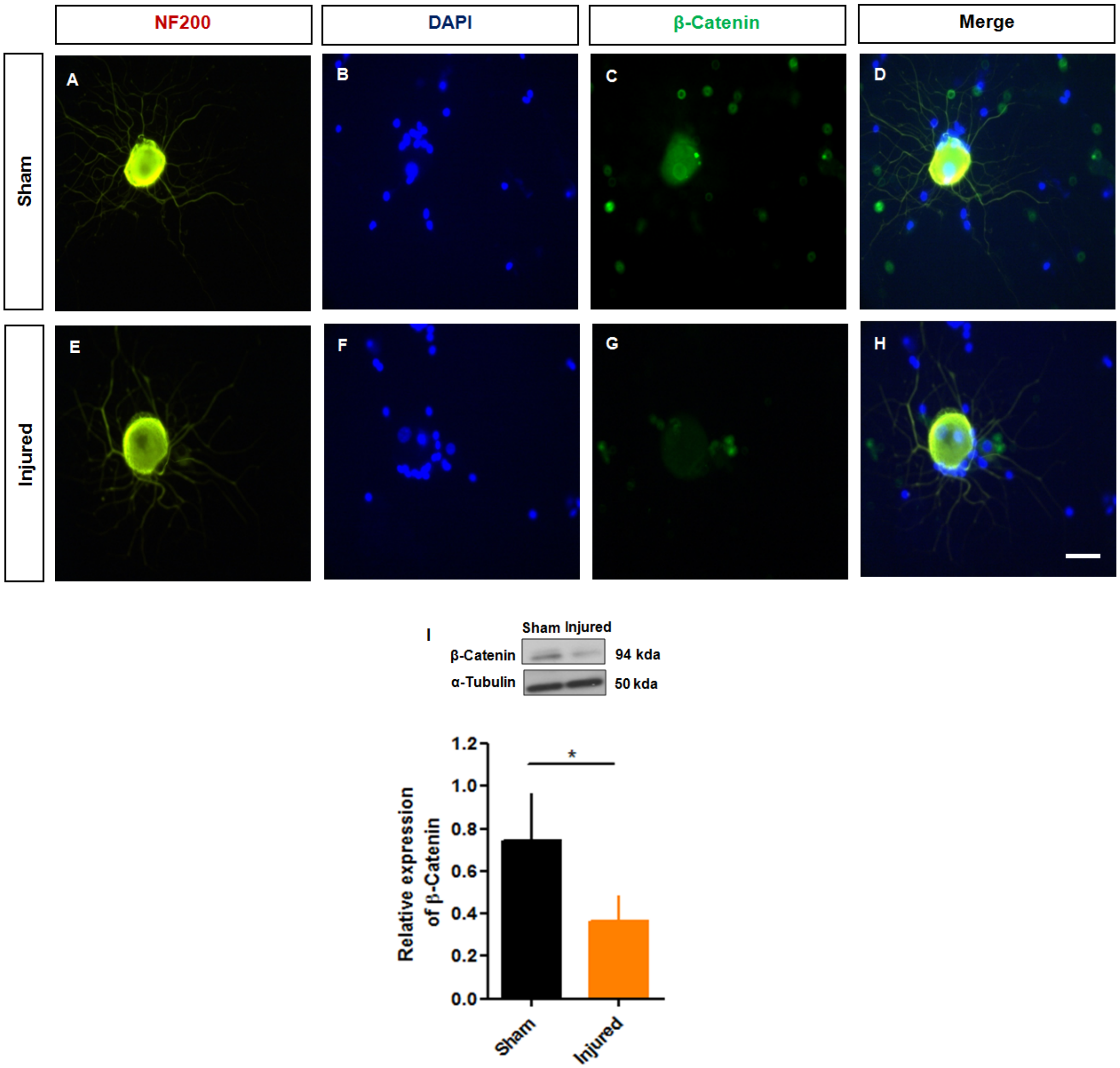

Supplemental Figure 6.

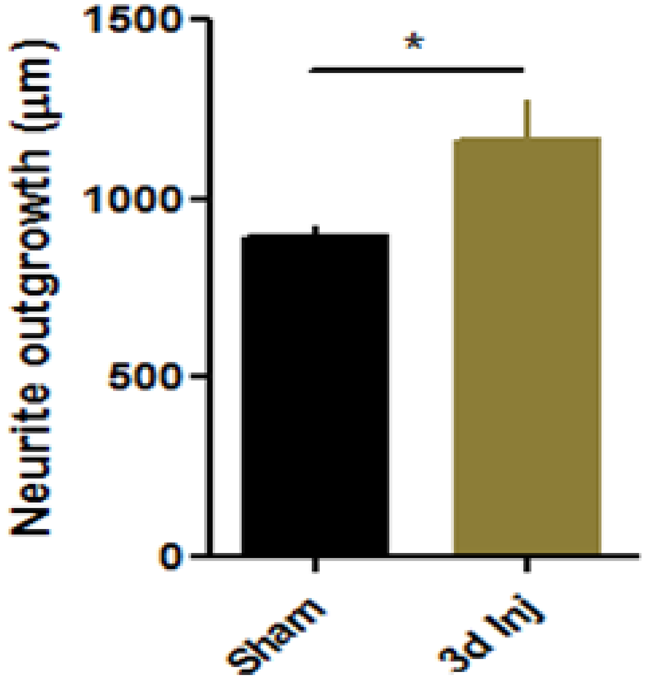

Supplemental Figure 7.

A

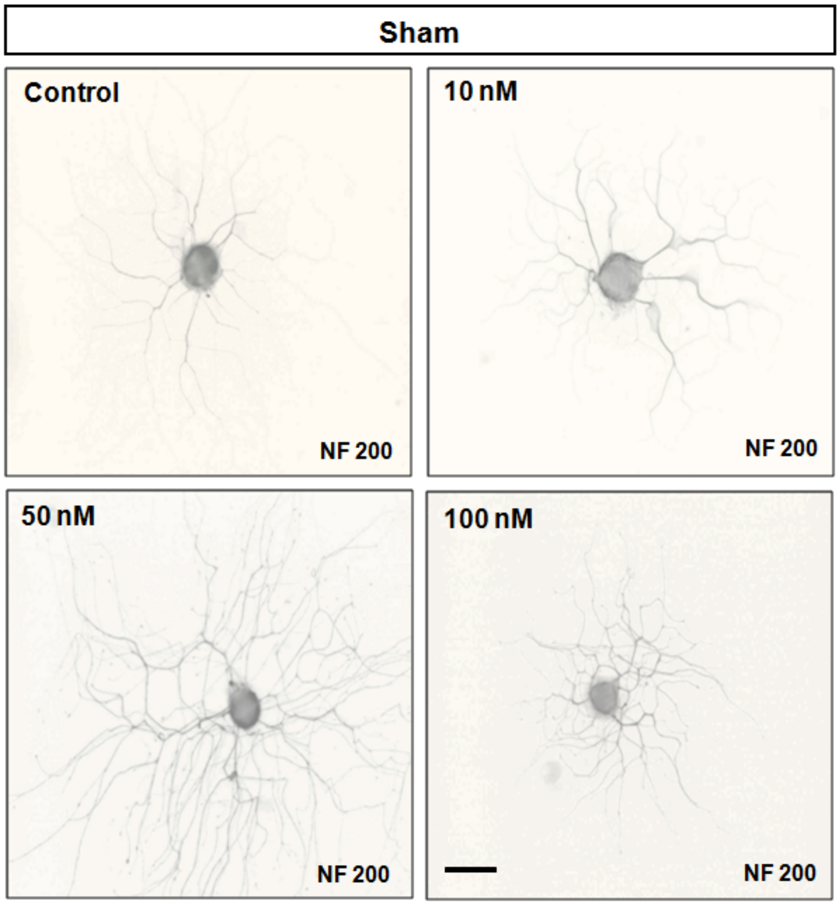

B

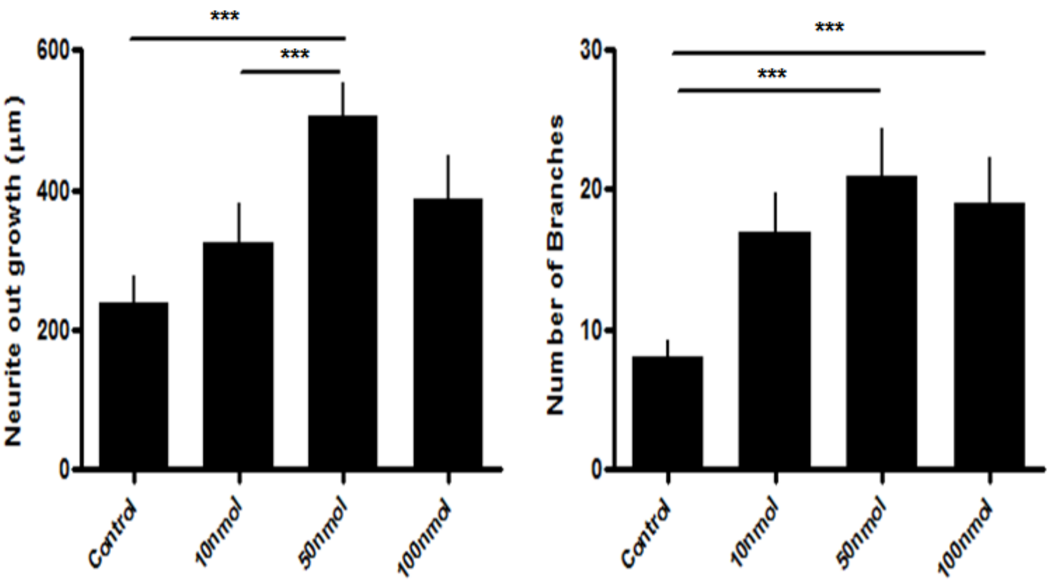

Supplemental Figure 8.

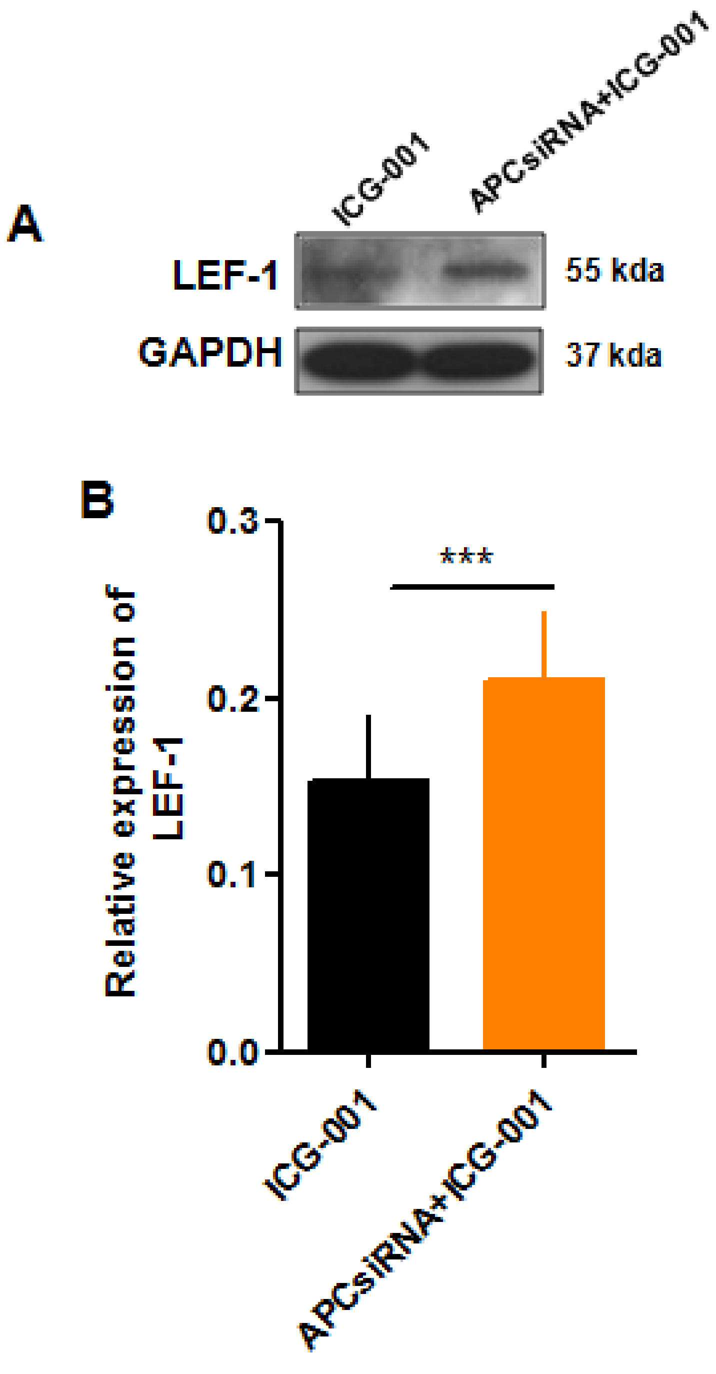

Supplemental Figure 9.

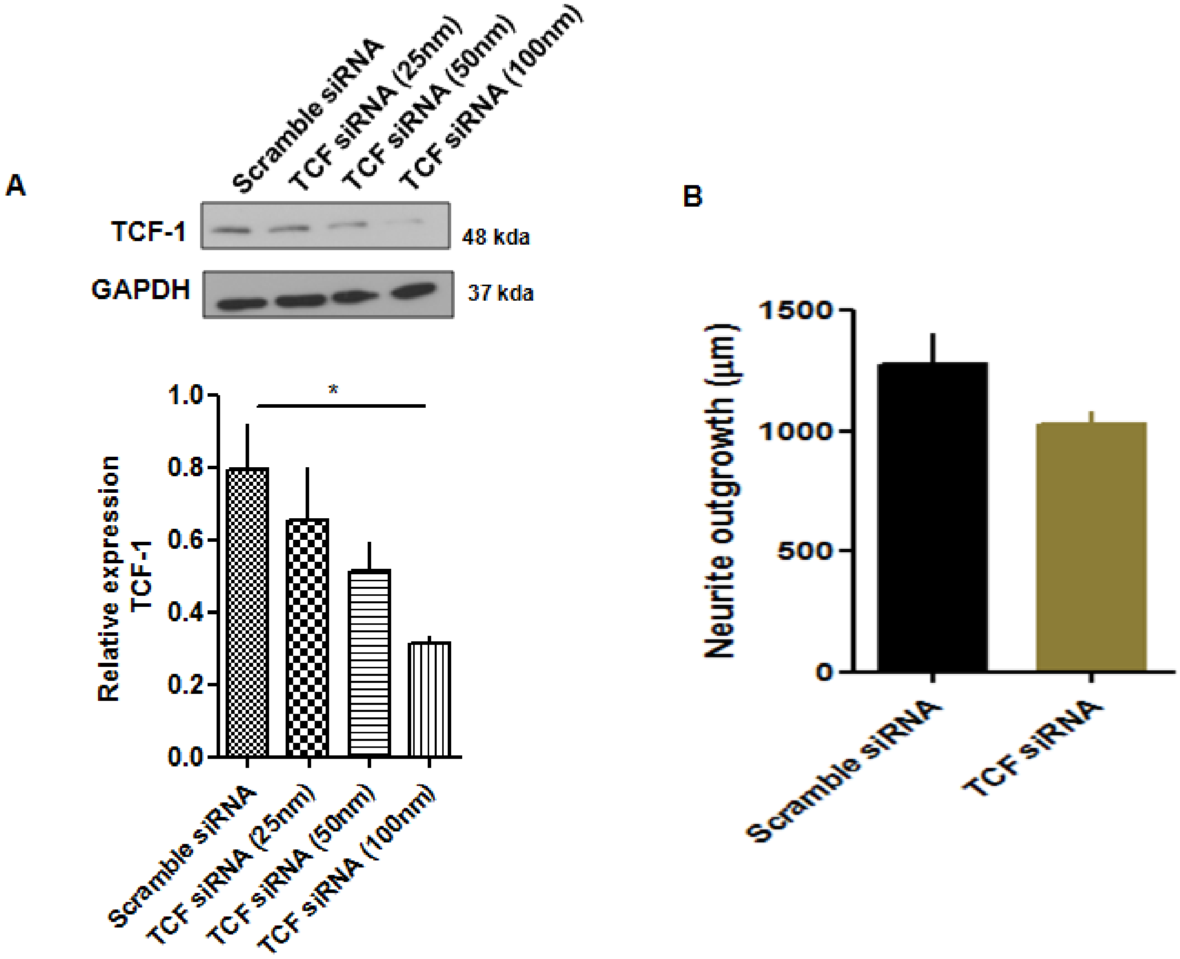

Supplemental Figure 10.

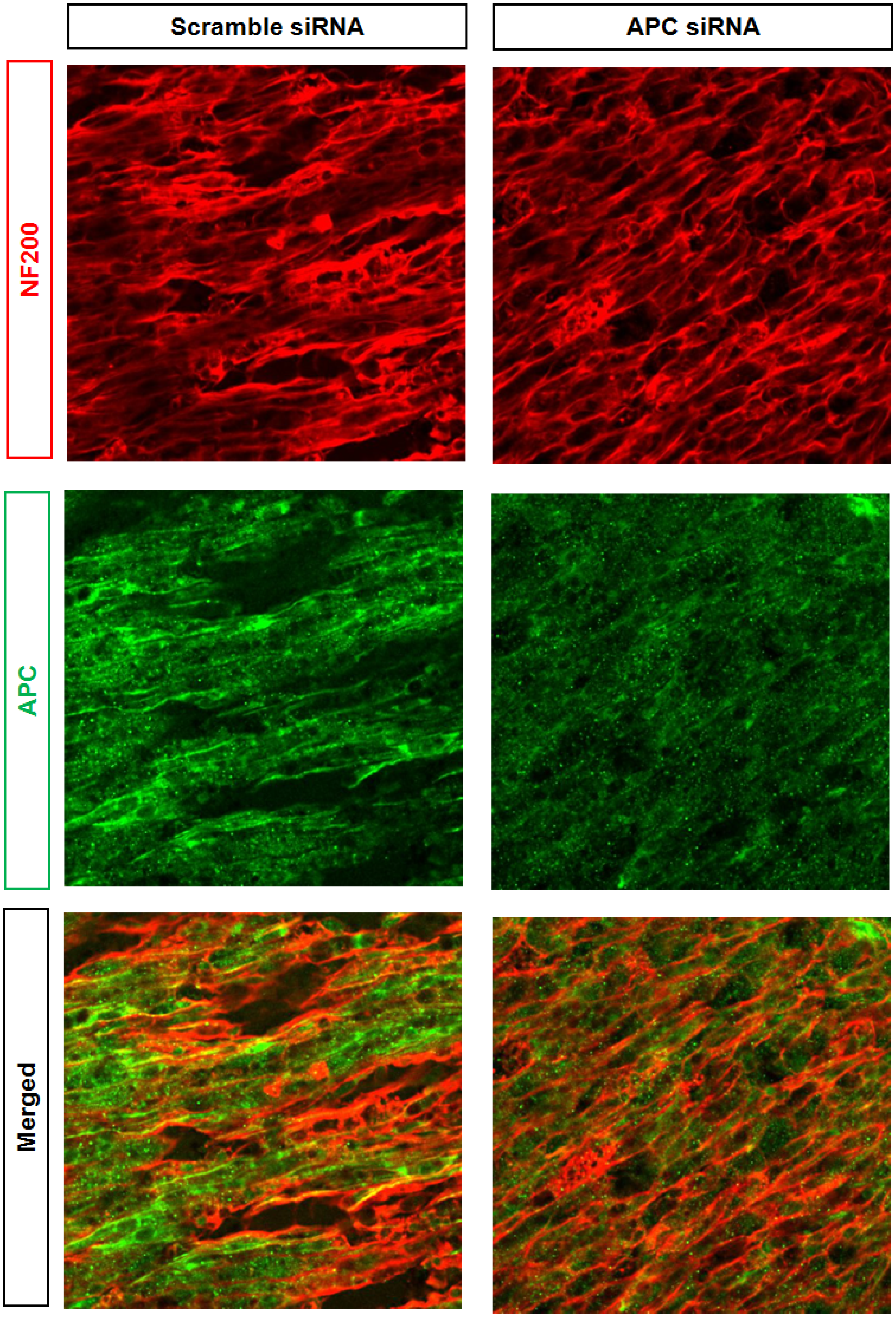

Supplemental Figure 11.

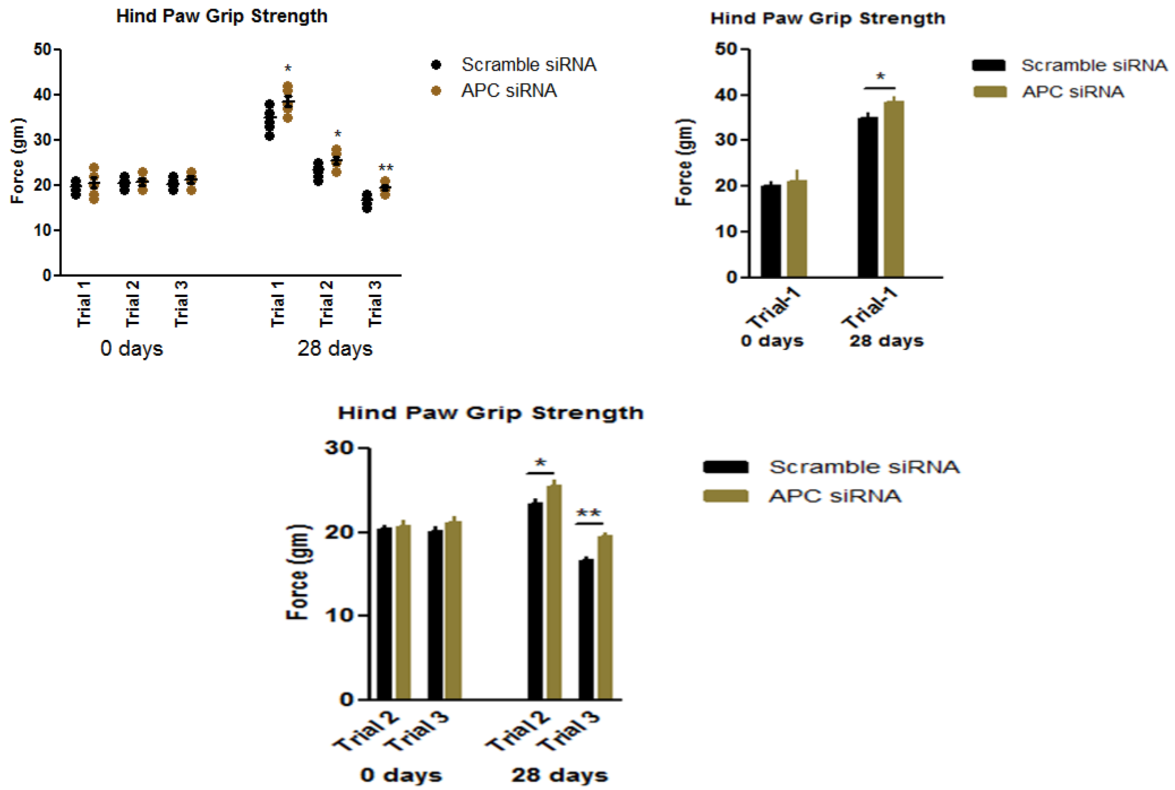

Supplemental Figure 12.

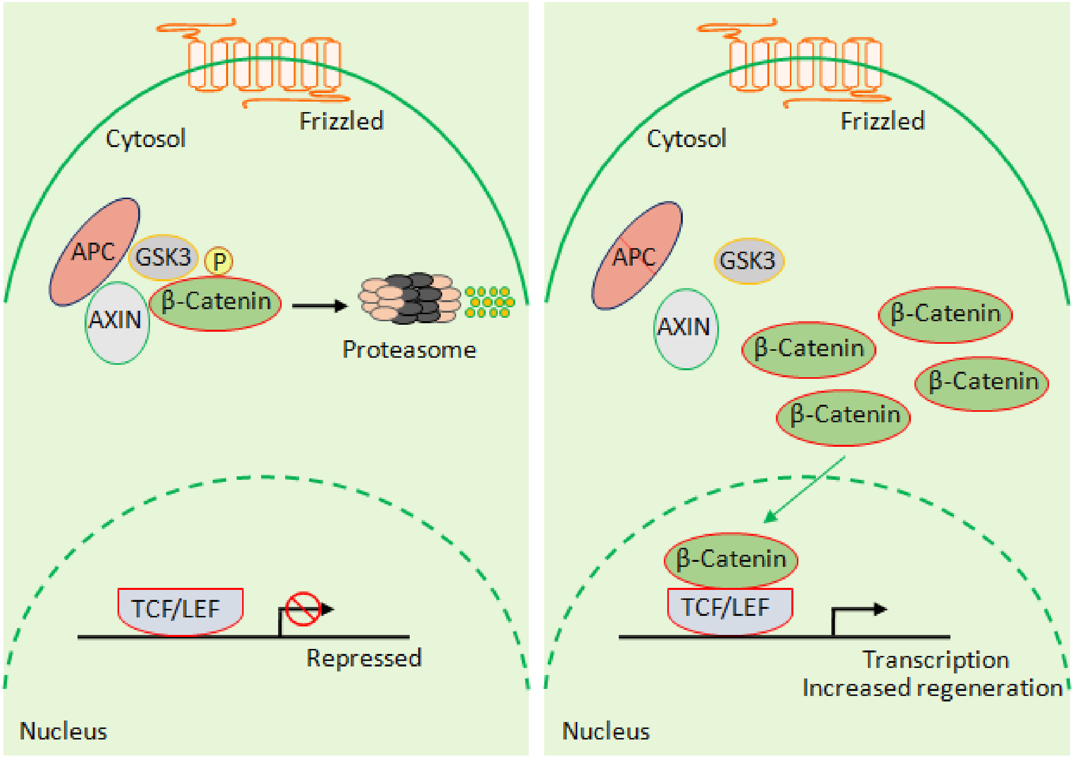

Supplemental Figure 13.

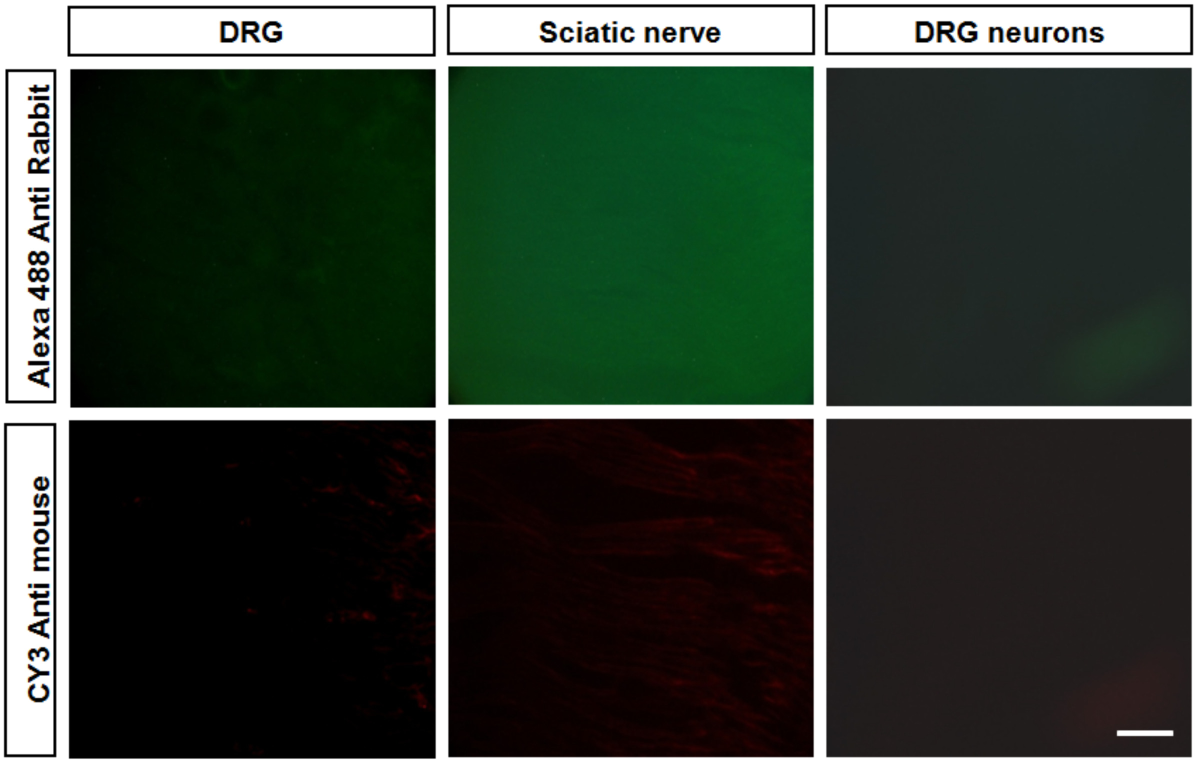

Supplement: Supplementary file 1 — Supplementary Information [file 41598_2018_31167_MOESM1_ESM.pdf]
